# Prokinetics for the treatment of functional dyspepsia: an updated systematic review and network meta-analysis

**DOI:** 10.1186/s12876-023-03014-9

**Published:** 2023-10-31

**Authors:** Qingqing Qi, Nana Wang, Han Liu, Yanqing Li

**Affiliations:** 1 Department of Gastroenterology, Cheeloo College of Medicine, Qilu Hospital, Shandong University, Jinan, Shandong Province 250012 People’s Republic of China; 2 Department of Radiation Oncology, Cheeloo College of Medicine, Qilu Hospital, Shandong University, Jinan, Shandong Province 250012 People’s Republic of China

**Keywords:** Prokinetics, Treatment, Functional dyspepsia

## Abstract

**Background:**

Since the previous network meta-analysis assessing the efficacy of prokinetics for functional dyspepsia (FD), there have been a number of new studies and cinitapride is a new prokinetic agent for FD. This updated meta-analysis aimed to explore the efficacy and safety of prokinetics for FD.

**Methods:**

An updated study search in Pubmed, EMBASE, Cochrane Library and Web of Science was conducted in literatures published from July 2015 to March 2023. Randomized controlled trials investigating the use of prokinetics in adult FD patients were included. The primary outcome was the total efficacy rate and the secondary outcome was adverse events. A Bayesian network meta-analysis was performed using R software.

**Results:**

A total of 28 studies were included. Network meta-analysis showed that metoclopramide had a higher total efficacy rate than mosapride (OR: 3.53, 95%CI: 1.70–7.47), domperidone (OR: 2.29, 95%CI: 1.16–4.63), itopride(OR: 2.77, 95%CI: 1.41–5.59), acotiamide(OR: 2.63, OR: 1.33–5.36), and placebo(OR: 5.68, 95%CI: 2.98–11.10), however similar to cinitapride (OR: 1.62, 95%CI: 0.75–3.53). Cinitapride had a higher total efficacy rate than mosapride (OR: 2.18, 95%CI: 1.16–4.14) and placebo (OR: 3.52, 95%CI: 2.01–6.24). Cinitapride had lower risk of total adverse events than domperidone. There was no difference in the risk of drug-related adverse events between the prokinetics.

**Conclusions:**

Metoclopramide and cinitapride may have a better efficacy than other prokinetics in the treatment of FD, and cinitapride may have a lower risk of total adverse events. Further studies using uniform definitions or validated tools to measure the total efficacy rate are needed.

**Supplementary Information:**

The online version contains supplementary material available at 10.1186/s12876-023-03014-9.

## Introduction

Functional dyspepsia (FD) is a prevalent gastrointestinal disorder of the gastroduodenal region that presents with upper abdominal symptoms unexplained by the presence of organic disease [1]. FD is classified into two subtypes: epigastric pain syndrome (EPS) and postprandial distress syndrome (PDS) based on the predominant symptom pattern. Motility disturbance is considered a component of the pathogenesis of FD, and a number of clinical trials have investigated the therapeutic effects of different kinds of prokinetics used for the treatment of FD [1].

A previous network meta-analysis assessed the comparative efficacy of six prokinetic agents for the treatment of FD [2]. However, since that publication, there have been a number of new studies, and cinitapride, a new kind of prokinetic agent, is currently considered an additional drug of choice for the treatment for FD [3, 4].

Therefore, our objectives were to carry out an up-to-date network meta-analysis to explore the efficacy and safety of prokinetics for managing FD.

## Methods

This is an updated systematic review of the published review “Prokinetics For The Treatment of Functional Dyspepsia: Bayesian Network Meta-analysis” [2]. An updated search was carried out in accordance with the Preferred Reporting Items for Systematic Reviews and Meta-analyses (PRISMA) extension statement [5, 6]. (Supplementary file 3, PRISMA NMA checklist)

### Literature search

An updated search in Pubmed, EMBASE, Cochrane Library, and Web of Science was conducted in literatures published from July 2015 to March 2023 without limitations on language or document type. Search strategies used in all databases are described in detail (Supplementary file 1, Search strategy). Reference lists of the included studies and relevant systematic reviews were reviewed to identify any additional papers. Clinicaltrials.gov and WHO trials registry were also searched for registered trials. We also contacted experts in this field to identify additional studies.

### Study selection

Two reviewers independently screened studies by viewing titles and abstracts. All potentially relevant citations were requested and inspected in detail using the full-text version. Disagreements were resolved by discussion with assistance from a third party, if necessary. A PRISMA flow diagram was constructed to show the full study-selection process.

Studies were selected if they met inclusion criteria: (1) Adult patients (at least 18 years old) diagnosed with symptoms of FD as defined by the original studies, (2) Randomized controlled trials (RCTs), (3) Treatment regimens that included drugs listed below: 1) Metoclopramide (Maxolon, Rimetin, Primperan, Reglan, Cerucal, clopamon, clopram, degan, emperal, imperan, metamide, metagliz, metozolv, pulin and terperan), 2) Mosapride, 3) Domperidone (Domperidon, Domidon, Gastrocure, Motilium), 4) Itopride (itopride, ganaton), 5) Acotiamide, 6) Cinitapride (Cidine, cinitaprid, Blaston), 7) Placebo. We only included single use of the above-listed drugs. There were no limitations on dosage, frequency time, treatment duration, and method of administration.

Exclusion criteria were: (1) other diseases of the upper gastrointestinal tract and upper abdominal organs that may present with similar dyspeptic symptoms (gastrointestinal malignancy, peptic ulcer, liver, gallbladder, and pancreatic disease), (2) studies that did not report eligible outcome data and studies that did not provide access to a full report. Moreover, we excluded trimebutine as it is not classified as a prokinetic drug.

### Data extraction

For each study, the following information was extracted by two independent reviewers: first author’s name, year of publication, country, diagnosis, sample sizes at random, sex and age of patients, intervention, drugs, dosage, effect size of the intervention and control groups, data and definition of outcome, and measurement time. Disagreements were resolved by discussion with assistance from a third party, if necessary. If multiple publications were reported for the same study, we extracted all data from the companion studies and removed the duplicated data.

### Quality assessment / risk of bias analysis

Two reviewers independently assessed the risk of bias of the included studies. We assessed each domain for risk of bias according to the standard criteria outlined in the Cochrane Handbook [7], including random sequence generation, allocation concealment, blinding of participants and personnel, blinding of outcome assessment, incomplete outcome data, selective outcome reporting, and other bias. Disagreements were resolved by discussion with assistance from a third party, if necessary.

### Outcome assessment

The primary outcome was the therapeutic efficacy (total efficacy rate). The secondary outcome was adverse events, including total adverse events, drug-related adverse events, and specific adverse events.

### Statistical analysis

As the efficacy is a representative of dichotomous outcomes, odds ratios (ORs) and 95% confidence intervals (CIs) were used as outcome measures. It could be simply explained that the experimental group exhibited a significantly higher efficacy compared to the control group when the ORs and its 95% CIs are more than 1. Where possible, we used the ORs based on an intention-to-treat (ITT) analysis of the population. A Bayesian network meta-analysis was performed using the network package (gemtc package) in R Studio 4.0 software. The fixed-effect models were used. The pooled estimation and the probability of identifying the most efficacious drug were obtained using the Markov Chains Monte Carlo method [8]. Evidence inconsistency and clinical similarity in patient characteristics and settings across trials were carefully assessed. Network geometry was performed using R software. Network geometry used nodes to represent different interventions and edges to represent the head-to-head comparisons between interventions. The size of nodes and thickness of edges were associated with the number of patients receiving specific interventions and the number of included trials, respectively. The node split method was used to check for consistency in the network. Based on these results, we calculated the surface under the cumulative ranking curve (SUCRA), which is the converted value reflecting the probability of a treatment being the best according to the ranking of each treatment [9]. A higher SUCRA value indicates better therapeutic results based on the indirect comparison method [10].

## Results

### Description of studies

#### Results of study selection

**Fig. 1:**
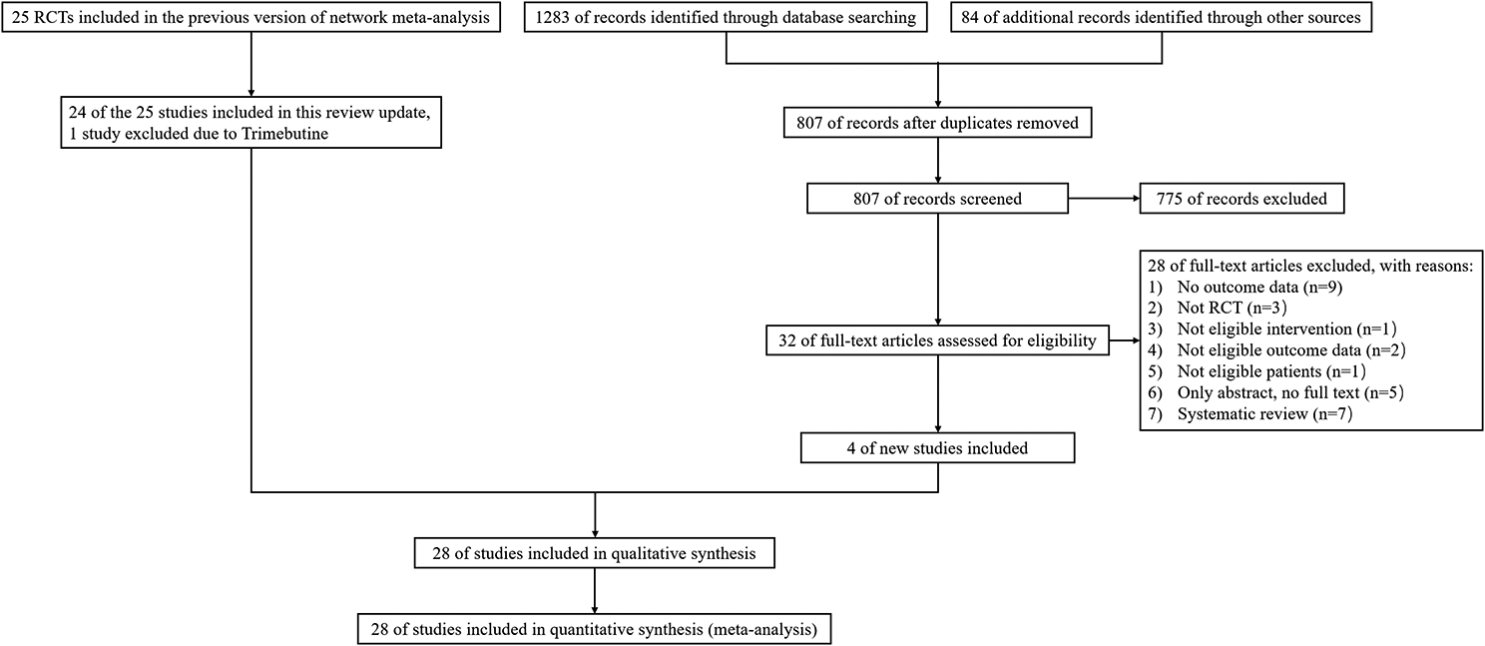
Flow diagram for identification of relevant studies

Figure 1 shows a flow diagram of the overall study selection process. The updated search retrieved 1283 references from electronic databases. Eighty-four references were identified from other sources. After deduplication, 807 references were screened by reviewing the title and abstract, and 775 references were excluded. Thirty-two full-text articles were assessed for eligibility and subsequently 28 were excluded with detailed reasons (further details are provided in Fig. 1). Four additional studies were included [3, 4, 11, 12]. Noticeably, this review included 24 studies from the original systematic review [2]. One study of trimebutine was excluded. Therefore, a total of 28 studies, and comparisons of 6 prokinetic drugs versus placebo were included in the final analysis. Among 28 RCTs, two RCTs (Matsueda 2010-study 1 and Matsueda 2010-study 2) were reported in one reference [13].

#### Characteristics of included studies

A total of 28 RCTs involving 5,790 participants met the inclusion criteria for this review, with sample sizes ranging from 16 to 892 (Supplementary Table 1).

##### Characteristics of participants

There were nine studies from China with 1,665 participants [3, 14–21], five studies from Belgium with 269 participants [22–26], one study from Germany with 548 participants [27], two studies from India with 280 participants [28] [12], six studies from Japan with 1778 participants [11, 13, 29–31], one study from Korea with 28 participants [32], one study from Denmark, Germany, France, Sweden, and the UK, with 566 participants [33], one study from Spain with 20 participants [4], and two studies from the US with 636 participants [34, 35].

The age of the participants ranged from 18 to 94 years; there were 1,951 males and 3,331 females. Three studies did not report the sex of 508 participants [18, 21, 23]. Most of the participants (n = 5,510) were diagnosed with FD, while the remaining 280 participants had postprandial distress syndrome, which was also classified as FD [12, 19].

##### Characteristics of interventions

In total, we included 28 RCTs with the interventions of metoclopramide, cinitapride, mosapride, domperidone, itopride, acotiamide, and placebo in this systematic review (Supplementary Table 1).

Six studies compared acotiamide with placebo [11, 13, 29–31], two studies reported in one report compared different dosages of acotiamide [13]. One study compared cinitapride with domperidone [3], and one study compared cinitapride with metoclopramide [14]. Six studies compared domperidone with placebo [22–26, 35]. Six studies compared itopride with domperidone [14, 16–18, 20, 21] and two studies compared itopride with placebo [27, 34]. Seven studies compared mosapride with domperidone [15], itopride [28], acotiamide [12] and placebo [19, 24, 32, 33]. The treatment duration ranged from 2 to 8 weeks. All the prokinetic agents were orally administered, and the detailed dosage, duration of treatment, and characteristics of the enrolled studies are shown in Supplementary Table 1.

##### Characteristics of outcomes: total efficacy rate

The definitions of these outcomes are described in the Supplementary Table 2. Eighteen studies reported the total efficacy rate [3, 4, 11–13, 17, 19–21, 23, 26, 27, 30, 31, 33–35]. Three studies measured the total efficacy rate using a reduction in symptom score and the response rate by subjects global assessment of overall treatment efficacy (OTE) questionnaire [12, 13, 30], Patient’s Global Symptomatic Improvement (PGSI) [31] or responder rate based on FD score [11]. Five studies reported the early satiation effective rate, which was measured by the clinical symptom scores [14–16, 18, 21]. The remaining studies did not report a definition of total efficacy.

##### Characteristics of outcomes: adverse events

Fifteen studies reported adverse events [3, 12–14, 16, 18–21, 29–31, 33, 35]. However, two studies did not report data [31, 33]. Three studies reported counts of adverse events [13, 30], six studies reported total adverse events [3, 12, 18–21], four studies reported drug-related adverse events [3, 14, 30, 35], and five studies reported specific adverse events [3, 16, 20, 29, 35]. Details regarding specific adverse events are described in Supplementary Table 3.

### Risk of bias in included studies

Details of the “Risk of bias” assessments are presented in the “Risk of bias” graph (Supplementary Fig. 1) and summary (Supplementary Fig. 2).

#### Random sequence generation

Fourteen studies reported adequate random sequence generation and were rated as low risk of bias. The methods used to generate the allocation sequence were random number tables and computer-generated programs. The remaining fourteen studies did not report sufficient information about random sequence generation; therefore, they were rated as unclear risk of bias in this domain [3, 4, 14, 16–18, 21, 24, 26, 29, 31–33, 35].

#### Allocation concealment

Thirteen studies reported the method of allocation concealment and were rated as low risk of bias in this domain [13, 15, 17, 22, 23, 25–30, 34]. Fifteen studies did not report sufficient information and were therefore rated as unclear risk of selection bias in this domain [3, 4, 11, 12, 14, 16, 18–21, 24, 31–33, 35].

#### Blinding of participants and personnel

Eighteen studies reported that the participants were blinded to the treatment, provided details of the blinding procedure, and were rated as low risk of bias [3, 11, 12, 15, 17, 18, 20–29, 31, 34]. Nine studies provided insufficient information to assess bias in this domain and were rated as unclear risk of detection bias [4, 13–15, 19, 30, 33, 35]. One study [32] was rated as high risk of bias.

#### Blinding of outcome assessment

Thirteen studies reported that the outcome assessors were blinded to the treatment and were rated as low risk of bias [15, 20–29, 31, 34]. The remaining fourteen studies provided insufficient information to assess bias in this domain and were rated as unclear risk of bias [4, 11–14, 16–19, 26, 30, 33, 35]. One study was rated as high risk of bias in this domain because two active drugs-cinitapride and domperidone-were compared, without the use of a placebo [3].

#### Incomplete outcome data

Twenty-five studies reported a low attrition rate (ranging from 4 to 10%), and the attrition rate was similar between the intervention and control groups. Therefore, these studies were rated as low risk of bias. Three studies provided insufficient information to assess bias in this domain and were rated as unclear risk of bias [4, 12, 31]. Six studies analyzed the data derived from the ITT population [3, 15, 21, 27, 30, 33].

#### Selective reporting

Protocols from five studies were available [3, 11, 13, 29]. All outcomes predefined in the protocol were mentioned in the methods and were reported in the final study report. Overall, the risk of reporting bias was low in the included studies.

#### Other potential sources of bias

We did not identify other potential sources of bias in twenty-four studies [4, 13–26, 28–35]. Four studies were funded by a pharmaceutical company but did not state whether the funder participated in the study process. Therefore, the potential bias is unclear [3, 11, 12, 27].

### Effects of interventions

#### Total efficacy rate of prokinetics in FD

A network plot of the total efficacy rate is presented in Supplementary Fig. 3. The biggest node was placebo, which involved 1,488 participants from 17 arms, followed by domperidone with 965 participants from 14 study arms, itopride with 955 participants from 9 study arms, acotiamide with 733 participants from 6 study arms, mosapride with 446 participants from 6 study arms, cinitapride with 210 participants from 2 study arms, and metoclopramide with 88 participants from 2 study arms.

**Fig. 2:**
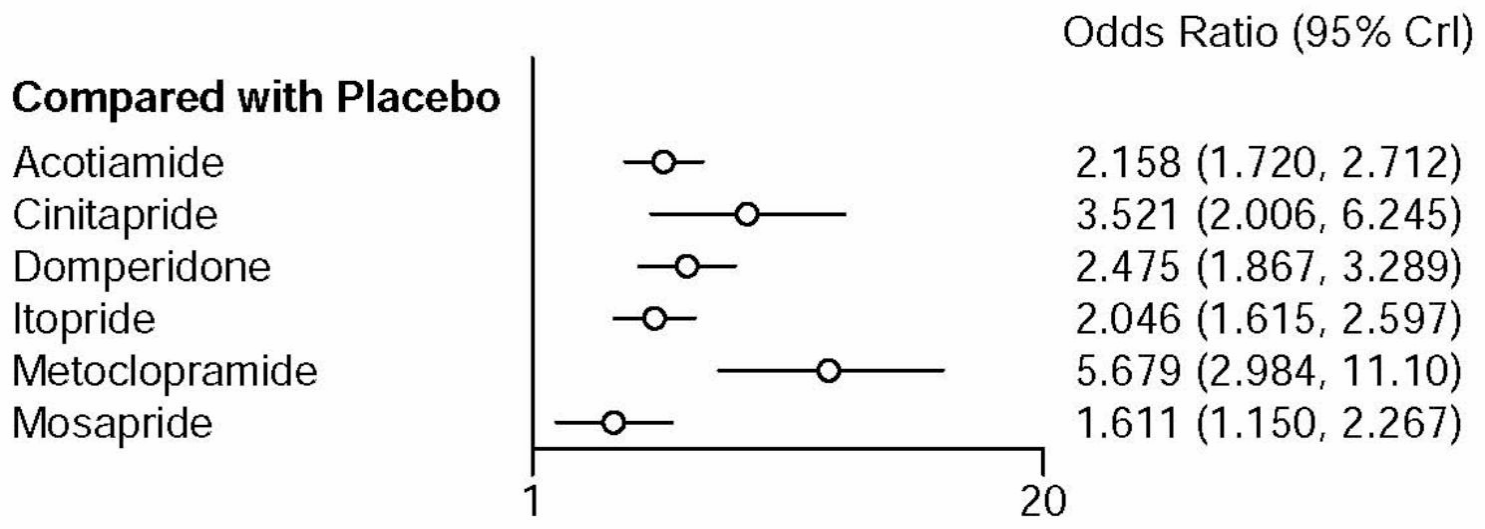
Forest plot of total efficacy rate between prokinetics and placebo

Network meta-analysis showed that cinitapride, mosapride, domperidone, metoclopramide, itopride, and acotiamide had a higher total efficacy rate than placebo (Fig. 2).

Table 1 describes the network meta-analysis league table of each treatment regimen. Metoclopramide showed a higher total efficacy rate than mosapride (OR: 3.53, 95%CI: 1.70–7.47), domperidone (OR: 2.29, 95%CI: 1.16–4.63), itopride (OR: 2.77, 95%CI: 1.41–5.59), acotiamide (OR: 2.63, OR: 1.33–5.36). However, there was no significant difference between metoclopramide and cinitapride (OR: 1.62, 95%CI: 0.75–3.53). Furthermore, cinitapride had a higher total efficacy rate than mosapride (OR: 2.18, 95%CI: 1.16–4.14), domperidone had a higher total efficacy rate than mosapride (OR: 1.54, 95%CI: 1.04–2.29). There was no significant difference in the total efficacy rate between the other prokinetics.

**Table 1:** League table of each treatment regimen on total efficacy rate

Acotiamide	1.63 (0.89, 3.02)	1.15 (0.80, 1.64)	0.95 (0.68, 1.31)	**2.63 (1.33, 5.36)**	0.75 (0.50, 1.11)	**0.46 (0.37, 0.58)**
0.61 (0.33, 1.13)	Cinitapride	0.70 (0.42, 1.18)	0.58 (0.33, 1.02)	1.62 (0.75, 3.53)	**0.46 (0.24, 0.87)**	**0.28 (0.16, 0.50)**
0.87 (0.61, 1.25)	1.42 (0.85, 2.40)	Domperidone	0.83 (0.64, 1.06)	**2.29 (1.16, 4.63)**	**0.65 (0.44, 0.97)**	**0.40 (0.30, 0.54)**
1.06 (0.76, 1.46)	1.72 (0.98, 3.03)	1.21 (0.94, 1.55)	Itopride	**2.77 (1.41, 5.59)**	0.79 (0.53, 1.15)	**0.49 (0.39, 0.62)**
**0.38 (0.19, 0.75)**	0.62 (0.28, 1.34)	**0.44 (0.22, 0.86)**	**0.36 (0.18, 0.71)**	Metoclopramide	**0.28 (0.13, 0.59)**	**0.18 (0.09, 0.34)**
1.34 (0.90, 1.99)	**2.18 (1.16, 4.14)**	**1.54 (1.04, 2.29)**	1.27 (0.87, 1.88)	**3.53 (1.70, 7.47)**	Mosapride	**0.62 (0.44, 0.87)**
**2.16 (1.72, 2.71)**	**3.52 (2.01, 6.24)**	**2.47 (1.87, 3.29)**	**2.05 (1.61, 2.06)**	**5.68 (2.98, 11.1)**	**1.61 (1.15, 2.27)**	Placebo

Notes: Odds ratio with 95% credible interval is described in each column. The odds ratio means prokinetic agent in the top left column comparing with prokinetic agent in the lower right column. Statistical validity is guaranteed when the 95% credible interval does not include 1, which is highlighted in bold

The Nodesplit inconsistency test of the total efficacy rate showed a high inconsistency between domperidone and mosapride, itopride and mosapride, itopride and domperidone, domperidone and placebo, and itopride and placebo (Supplementary Fig. 4).

Pairwise meta-analysis showed similar findings to the network meta-analysis except that the total efficacy rate between mosapride and domperidone, mosapride and itopride, mosapride and placebo. Pairwise meta-analysis showed that there was no difference on total efficacy rate between mosapride and domperidone or placebo, while mosapride had lower total efficacy rate than itopride (Supplementary Fig. 5).

The treatment ranking probability showed the total efficacy rate of metoclopramide (97.9%) ranked first, followed by cinitapride (82.1%), domperidone (63.2%), acotiamide (47.3%), itopride (39.3%), mosapride (20.1%), and placebo (0.0%) (Supplementary Table 4).

#### Adverse events

##### Total adverse events

The network plot of total adverse events was presented in Supplementary Fig. 6. The biggest node of total adverse events was domperidone with 526 participants from 4 study arms, followed by itopride with 334 participants from 3 study arms, cinitapride with 191 participants from 1 study arm, mosapride with 142 participants from 2 study arms, acotiamide with 108 participants from 1 study arm and placebo with 30 participants from 1 study arm (Supplementary Fig. 6).

**Fig. 3:**
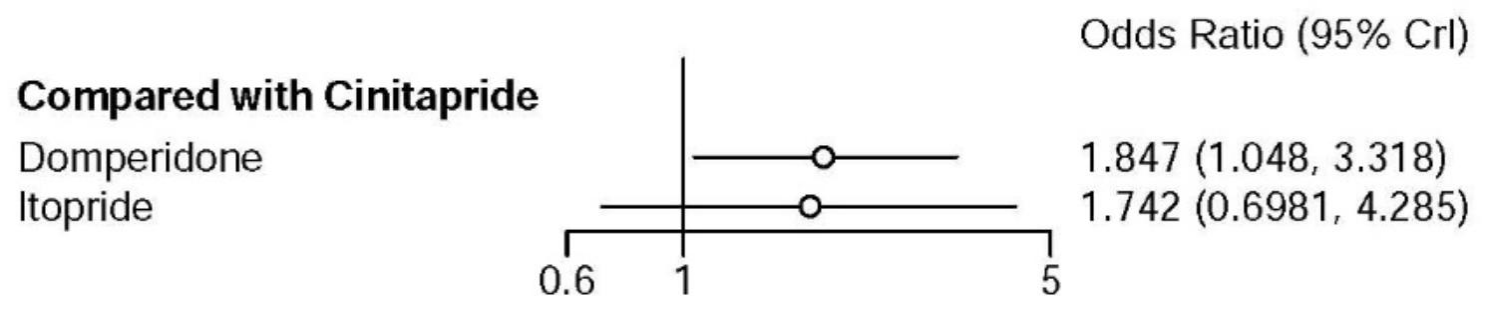
Forest plot of total adverse events when cinitapride compared with domperidone and itopride

Network meta-analysis showed there was no significant difference in the risk of total adverse events when cinitapride compared with itopride, however, domperidone had higher risk of total adverse events than cinitapride (OR: 1.85, 95%CI: 1.05–3.32) (Fig. 3).

Table 2 describes the league table of total adverse events associated with each treatment regimen. There was no significant difference in the risk of total adverse events between cinitapride and itopride as well as domperidone and itopride.

**Table 2:** League of each treatment regimen on total adverse events

Cinitapride	**1.85 (1.05, 3.32)**	1.74 (0.70, 4.29)
**0.54 (0.30, 0.95)**	Domperidone	0.94 (0.46, 1.87)
0.57 (0.23, 1.43)	1.06 (0.54, 2.15)	Itopride

Notes: Odds ratio with 95% credible interval is described in each column. The odds ratio means prokinetic agent in the top left column comparing with prokinetic agent in the lower right column. Statistical validity is guaranteed when the 95% credible interval does not include 1, which is highlighted in bold

The Nodesplit plot of total adverse events rate could not be conducted due to insufficient data.

The findings of the pairwise meta-analysis were consistent with those of the network meta-analysis (Supplementary Fig. 7). Direct comparison results from individual studies [19] showed that there was no difference in the risk of total adverse events between mosapride and placebo [19] or acotiamide [12].

The treatment ranking probability showed that as for the safety of total adverse events, cinitapride (93.5%) ranked highest, followed by itopride (34.1%) and domperidone (22.5%) (Supplementary Table 5).

##### Drug-related adverse events

For drug-related adverse events, the biggest node was domperidone with 241 participants from 3 study arms, followed by cinitapride with 191 participants from 1 study arm, itopride with 40 participants from 1 study arm, and placebo with 7 participants from 1 study arm (Supplementary Fig. 8).

**Fig. 4:**
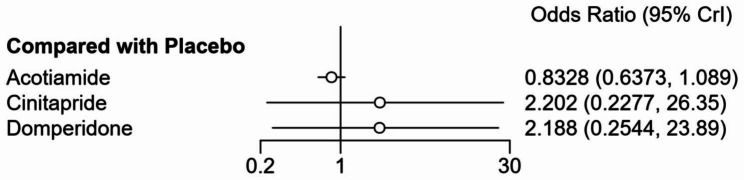
Forest plot of drug-related adverse events rate when cinitapride, domperidone, acotiamide compared with placebo

Network meta-analysis showed there was no significant difference in the risk of drug-related adverse events when cinitapride, domperidone, acotiamide were compared with placebo (Fig. 4).

Table 3 describes the league table of drug-related adverse events associated with each treatment regimen. There was no difference in the rate of drug-related adverse events between cinitapride, domperidone, itopride, acotiamide, and placebo.

**Table 3:** League of each treatment regimen on drug-related adverse events

Cinitapride	1.00 (0.47, 2.07)	0.04 (0, 6421.09)	0.37 (0.03, 3.67)	0.45 (0.04, 4.32)
1.00 (0.48, 2.11)	Domperidone	0.04 (0, 6038.07)	0.38 (0.03, 3.38)	0.46 (0.04, 4.02)
27.34 (0, 148817954.82)	27.3 (0, 143585383.88)	Itopride	10.25 (0, 60877502.63)	12.29 (0, 72591299.15)
2.67 (0.27, 32.42)	2.64 (0.3, 29.04)	0.10 (0, 19807.02)	Acotiamide	1.20 (0.91, 1.57)
2.23 (0.23, 27)	2.19 (0.25, 23.8)	0.08 (0, 16299.35)	0.83 (0.64, 1.09)	Placebo

Notes: Odds ratio with 95% credible interval is described in each column. Prokinetic agent in the top left means better efficacy and statistical validity is guaranteed when the 95% credible interval does not include 1

The Nodesplit plot of drug-related adverse events rate could not be conducted due to insufficient data.

The ranking probability analysis showed that for the safety of drug-related adverse events, domperidone (69.1%) ranked highest, followed by cinitapride (68.7%), placebo (51.2%), itopride (32.8%), and acotiamide (28.2%) (Supplementary Table 6). Direct comparison results from individual studies showed there was no difference in the risk of drug-related adverse events between itopride and domperidone [14], cinitapride and domperidone [3], and placebo and domperidone [35].

##### Specific adverse events

The common specific adverse event (≥ 5%) associated with acotiamide was abnormal blood levels of prolactin (2/21) [29]. The common specific adverse events associated with domperidone were lower limb skin rash (2/20), mild lower abdominal pain (1/20), expressive galactorrhea (1/20) [16], drug-related constipation (1/9), drug-related expressive galactorrhea and bilateral breast tenderness (4/9), and drug-related hyperprolactinemia (9/9) [35]; and non-drug related AE (16/192) [3]. The common specific adverse events associated with itopride included lower limb skin rash (3/20) and mild lower abdominal pain (2/20) [16]. However, most adverse events were mild or moderate in severity. The placebo group reported adverse events similar to those that occurred in the prokinetics group (Supplementary Table 3).

## Discussion

This updated network meta-analysis included an additional four RCTs [3, 4, 11, 12] in comparison to the previously published meta-analysis [2]. The findings from this network meta-analysis indicated that the total efficacy rates of six prokinetic agents—metoclopramide, cinitapride, mosapride, domperidone, itopride, and acotiamide—were superior to that of the placebo. While there was no statistically significant difference in the total efficacy rate between metoclopramide and cinitapride, metoclopramide exhibited a significantly higher efficacy compared to the other four prokinetic treatments. Furthermore, cinitapride demonstrated a higher total efficacy rate than mosapride.

In our study we also calculated the SUCRA, which could reflect the probability of a treatment being the best according to the ranking of each treatment [9]. A higher SUCRA value indicates a higher probability of better treatment effect [10]. The ranking probability analysis indicated metoclopramide as the top-ranking treatment, followed by cinitapride, then domperidone, acotiamide, itopride, mosapride and placebo. When using SUCRA to evaluate the total adverse events and drug-related adverse events related to prokinetics, higher value means higher probability of a safer drug. However, the stability of these rankings should be interpreted cautiously, as they can be influenced by factors such as the number of included studies, the number of events, and the overall sample size.

The precise underlying pathogenesis of FD remains uncertain. Many contributing factors, including gastroduodenal motility abnormalities, visceral hypersensitivity, gastric acid, *Helicobacter pylori* infection, and psychosomatic influences, are believed to be implicated in the pathogenic process. Numerous clinical studies and meta-analyses have explored the effectiveness of therapies targeting the inhibition of visceral hypersensitivity, acid suppression, *Helicobacter pylori* eradication, and antipsychotic interventions for FD treatment. These studies have also conducted comparisons of the efficacy and adverse event profiles of various drugs [36–40]. Both the prior meta-analysis [2] and our current updated network meta-analysis have evaluated the therapeutic effects of distinct prokinetic agents for the treatment of FD. The objective of both of these meta-analyses is to offer clinicians additional evidence to aid in their selection of appropriate prokinetic agents. Our results confirmed that the six prokinetic agents included in the analysis were all significantly better than placebo. This revealed that prokinetic drugs should be effective in the treatment of FD. The treatment ranking probability showed that metoclopramide and cinitapride ranked the top two most effective drugs, suggesting that these two prokinetic agents may be the preferred drugs in FD treatment.

Prokinetic drug is a group of important therapy to improve the symptoms of FD, especially for FD patients with PDS. At present, various prokinetic agents have been used to treat FD. Cinitapride is a prokinetic agent that has dual effects of 5-HT receptor agonist and dopamine receptor antagonist. It has been proved that cinitapride can promote gastric emptying and motility, therefore, can be used in the treatment of motility related diseases such as dyspepsia, gastroesophageal reflux disease and so on [3]. Although clinical trials have been verified the effectiveness of cinitapride for FD [3, 14], the treatment status of cinitapride for FD compared with other prokinetic drugs remains unclear. Young Joo Yang conducted a meta-analysis comparing prokinetic agents for FD in 2017 and found that the treatment effect of metoclopramide, mosapride and domperidone is superior to itopride or acotiamide [2]. However, cinitapride was not included in the meta-analysis. In our update study, RCTs evidence relevant to cinitapride were included and a network meta-analysis was conducted. Results showed that cinitapride and metoclopramide were better than other prokinetic drugs for the treatment of FD. Our results are consistent with the results of a recently published meta-analysis conducted by Liang Liang and colleagues [41]. The authors compared the effects of different categories of drugs with different mechanisms for FD. The results showed that the antidepressant levosulpiride ranked the highest, followed by cinitapride ranked second among all drugs. Meanwhile, cinitabride was superior to other prokinetic and anti-acid agents [41].

However, the adverse events associated with the interventions have to be considered when selecting prokinetics. Result showed that cinitapride had lower risk of total adverse events than domperidone; however, there was no difference for total adverse events between acotiamide, mosapride and placebo. There was also no difference in drug-related adverse events between domperidone, acotiamide, cinitapride, and placebo. Therefore, considering both efficacy and safety, cinitapride seems the preferred prokinetic agents for the treatment of FD.

Meanwhile, clinicians should pay close attention to the risk of abnormal blood levels of prolactin when administering acotiamide, as well as to the development of lower limb skin rash, mild lower abdominal pain, expressive galactorrhea, constipation, and hyperprolactinemia when prescribing domperidone. Nonetheless, these results also reflect the rarity of adverse events reported in RCTs. Observational studies with a larger sample size are required for more precise estimates of the risk of adverse events.

Overall, the quality of evidence for the outcome of total therapeutic efficacy and adverse events was moderate. Low to moderate risk of bias were rated across seven domains of the risk of bias assessment tool. In half of the included studies (14/28), the methods used for random sequence generation and allocation concealment were not clearly reported. The attrition bias and reporting bias were low across the included studies. Some studies (6/28) reported they used an ITT analysis to deal with missing data. For potential other bias, four studies reported that the study was funded by pharmaceutical industry however the funder was not involved in the research process [3, 11, 12, 27].

This study was based on a previous systematic review [2], and an update literature search was performed. Our study identified two RCTs relevant to cinitapride, a new prokinetic agent. Therefore, our findings will provide more information for clinicians to use when making decisions.

The study selection process in our study includes trials with varying sample sizes, methodologies and study populations. The potential heterogeneity in study designs and patient characteristics could impact the validity and generalizability of the observed treatment effects and adverse event profiles, which should be considered when discussing the practical applicability of the study’s findings to real-world clinical practice. We have tried our best to mitigate the impact of heterogeneity in this study. First, the updated search strategy was developed by an information specialist, which helped identify a greater number of studies, and only RCTs investigating the use of prokinetics in adult FD patients were included in this study to reduce the potential heterogeneity in study designs. Second, two review authors independently selected and extracted data, ensuring the transparency of the review process and the accuracy of the network meta-analysis. Third, rigorous and normative statistical analysis were carried out in each step in this meta-analysis, which is important for controlling the impact of heterogeneity. The exact impact of heterogeneity might be evaluated by subgroup analysis. However, for the included 28 RCTs, subgroup analysis for different study designs and patient characteristics could only include limited studies for each subgroup and lead to unreliable results. Moreover, the main purpose of this updated meta-analysis is to explore the totally efficacy and safety of prokinetics for FD. Subgroup analysis for heterogeneity in study designs and patient characteristics would be performed in the future as soon as possible when much more studies would be carried out, especially when enough number of studies and data focusing on each subgroup factor would be published.

This study also has several limitations. First, in the network meta-analysis, there is a small number of studies and participants in the cinitapride and metoclopramide group, which may underestimate the effect of these two interventions. The inconsistency between domperidone and mosapride, itopride and mosapride, itopride and domperidone, domperidone and placebo, itopride and placebo may also compromise the robustness and reliability of the network meta-analysis. Moreover, the definition of the total efficacy rate varies across studies, and different measurement criteria may also influence the accuracy of the network meta-analysis. Lastly, the Asia limited marketing of a number of prokinetics is a relevant limitation which impact generalizability of findings.

## Conclusions

In conclusion, this network meta-analysis revealed metoclopramide and cinitapride may have a better total efficacy rate than other prokinetics, and cinitapride may have a lower risk of total adverse events. However, the results should be interpreted with caution due to insufficient data and the varied definitions of total efficacy rate. More RCTs comparing cinitapride and metoclopramide with placebo and other prokinetics are needed to better assess their efficacy. Further studies using uniform definitions or validated tools to measure total efficacy rate are also needed.

### Electronic supplementary material

Below is the link to the electronic supplementary material.

Supplementary Material 1

Supplementary Material 2

Supplementary Material 3

Supplementary Material 4

Supplementary Material 5

## Data Availability

The datasets used and/or analysed during the current study are available from the corresponding author on reasonable request.

## Abbreviations

FD: Functional dyspepsia
EPS: Epigastric pain syndrome
PDS: Postprandial distress syndrome
RCTs: Randomized controlled trials
PRISMA: Preferred Reporting Items for Systematic Reviews and Meta-analyses
ORs: Odds ratios
CIs: Confidence intervals
ITT: Intention-to-treat
SUCRA: The surface under the cumulative ranking curve
