# Supplementary material for: Prokinetics for the treatment of functional dyspepsia: an updated systematic review and network meta-analysis
Source: BMC Gastroenterol. 2023 Oct 31;23:370. doi: 10.1186/s12876-023-03014-9 (PMC10617220; doi:10.1186/s12876-023-03014-9)
Supplement: Supplementary file 1 — Supplementary Material 1 [file 12876_2023_3014_MOESM1_ESM.docx]

**Search strategy**

**PubMed**

(("functional dyspepsia"[tw] OR "functional dyspeptic"[tw] OR "functional dyspep*"[tw] OR "Dyspepsia/physiopathology"[mesh] OR "Postprandial distress"[tw] OR "postprandial discomfort"[tw] OR "Post prandial distress"[tw] OR "post prandial discomfort"[tw] OR "Epigastric pain syndrome"[tw]) AND ("Metoclopramide"[Mesh] OR Metoclopramide[tw] OR Maxolon[tw] OR Rimetin[tw] OR Primperan[tw] OR Reglan[tw] OR Cerucal[tw] OR clopamon[tw] OR clopram[tw] OR degan[tw] OR emperal[tw] OR imperan[tw] OR metamide[tw] OR metagliz[tw] OR metozolv[tw] OR pulin[tw] OR terperan[tw] OR "Trimebutine"[Mesh] OR Trimebutin*[tw] OR TM906[tw] OR "TM 906"[tw] OR Transacalm[tw] OR Polibutin[tw] OR Modulon[tw] OR Debridat[tw] OR cerekinon[tw] OR "mosapride"[Supplementary Concept] OR mosapride[tw] OR "AS 4370"[tw] OR AS4370[tw] OR tak370[tw] OR "tak 370"[tw] OR "Domperidone"[Mesh] OR Domperidon*[tw] OR Domidon[tw] OR Gastrocure[tw] OR Motilium[tw] OR Nauzelin[tw] OR biperidys[tw] OR costi[tw] OR nauzelin[tw] OR oroperidys[tw] OR tametil[tw] OR "itopride"[Supplementary Concept] OR itopride[tw] OR ganaton[tw] OR hsr803[tw] OR "hsr 803"[tw] OR "cinitapride"[Supplementary Concept] OR Cidine[tw] OR cinitaprid*[tw] OR Blaston[tw] OR "Placebos"[Mesh] OR Placebo*[tw] OR "Z 338"[Supplementary Concept] OR "Z 338"[tw] OR "acotiamide"[tw]) AND ("controlled clinical trial"[pt] OR "Controlled Clinical Trials as Topic"[MeSH] OR "Random Allocation"[MeSH] OR "Double-Blind Method"[MeSH] OR "single-blind method"[MeSH] OR "Double-Blind*"[tw] OR "single-blind*"[tw] OR "Triple-Blind*"[tw] OR "Control Groups"[MeSH] OR "cross-over studies"[MeSH] OR random*[tw] OR "Placebos"[mesh] OR placebo[tw] OR trial[tw] OR groups[tw] OR crossover[tw] OR cross-over[tw]) NOT ("Animals"[Mesh] NOT "Humans"[Mesh]))

**Embase**

(("functional dyspepsia".mp OR "functional dyspeptic".mp OR "functional dyspep*".mp OR "Postprandial distress".mp OR "postprandial discomfort".mp OR "Post prandial distress".mp OR "post prandial discomfort".mp OR "Epigastric pain syndrome".mp) AND ("Metoclopramide"/ OR Metoclopramide.mp OR Maxolon.mp OR Rimetin.mp OR Primperan.mp OR Reglan.mp OR Cerucal.mp OR clopamon.mp OR clopram.mp OR degan.mp OR emperal.mp OR imperan.mp OR metamide.mp OR metagliz.mp OR metozolv.mp OR pulin.mp OR terperan.mp OR "Trimebutine"/ OR Trimebutin*.mp OR TM906.mp OR "TM 906".mp OR Transacalm.mp OR Polibutin.mp OR Modulon.mp OR Debridat.mp OR cerekinon.mp OR "mosapride"/OR mosapride.mp OR "AS 4370".mp OR AS4370.mp OR tak370.mp OR "tak 370".mp OR "Domperidone"/ OR Domperidon*.mp OR Domidon.mp OR Gastrocure.mp OR Motilium.mp OR Nauzelin.mp OR biperidys.mp OR costi.mp OR nauzelin.mp OR oroperidys.mp OR tametil.mp OR "itopride"/OR itopride.mp OR ganaton.mp OR hsr803.mp OR "hsr 803".mp OR "cinitapride"/OR Cidine.mp OR cinitaprid*.mp OR Blaston.mp OR "Placebos"/ OR Placebo*.mp OR "Z 338"/OR "Z 338".mp OR "acotiamide".mp) AND (exp "controlled clinical trial"/ OR exp "Controlled Clinical Trial (Topic)"/ OR "Random Allocation"/ OR "Double Blind Procedure"/ OR "single blind procedure"/ OR "Double-Blind*".mp OR "single-blind*".mp OR "Triple-Blind*".mp OR "Control Group"/ OR "crossover procedure"/ OR random*.mp OR "Placebo"/ OR placebo.mp OR trial.mp OR groups.mp OR crossover.mp OR cross-over.mp) NOT (exp "Animals"/ NOT exp "Humans"/))

**Cochrane Library**

(("functional dyspepsia" OR "functional dyspeptic" OR "functional dyspep*" OR "Postprandial distress" OR "postprandial discomfort" OR "Post prandial distress" OR "post prandial discomfort" OR "Epigastric pain syndrome") AND ("Metoclopramide" OR Metoclopramide OR Maxolon OR Rimetin OR Primperan OR Reglan OR Cerucal OR clopamon OR clopram OR degan OR emperal OR imperan OR metamide OR metagliz OR metozolv OR pulin OR terperan OR "Trimebutine" OR Trimebutin* OR TM906 OR "TM 906" OR Transacalm OR Polibutin OR Modulon OR Debridat OR cerekinon OR "mosapride" OR mosapride OR "AS 4370" OR AS4370 OR tak370 OR "tak 370" OR "Domperidone" OR Domperidon* OR Domidon OR Gastrocure OR Motilium OR Nauzelin OR biperidys OR costi OR nauzelin OR oroperidys OR tametil OR "itopride" OR itopride OR ganaton OR hsr803 OR "hsr 803" OR "cinitapride" OR Cidine OR cinitaprid* OR Blaston OR "Placebos" OR Placebo* OR "Z 338" OR "Z 338" OR "acotiamide")):ti,ab,kw

**Web of Science**

TS=(("functional dyspepsia" OR "functional dyspeptic" OR "functional dyspep*" OR "Postprandial distress" OR "postprandial discomfort" OR "Post prandial distress" OR "post prandial discomfort" OR "Epigastric pain syndrome") AND ("Metoclopramide" OR Metoclopramide OR Maxolon OR Rimetin OR Primperan OR Reglan OR Cerucal OR clopamon OR clopram OR degan OR emperal OR imperan OR metamide OR metagliz OR metozolv OR pulin OR terperan OR "Trimebutine" OR Trimebutin* OR TM906 OR "TM 906" OR Transacalm OR Polibutin OR Modulon OR Debridat OR cerekinon OR "mosapride"OR mosapride OR "AS 4370" OR AS4370 OR tak370 OR "tak 370" OR "Domperidone" OR Domperidon* OR Domidon OR Gastrocure OR Motilium OR Nauzelin OR biperidys OR costi OR nauzelin OR oroperidys OR tametil OR "itopride"OR itopride OR ganaton OR hsr803 OR "hsr 803" OR "cinitapride"OR Cidine OR cinitaprid* OR Blaston OR "Placebos" OR Placebo* OR "Z 338"OR "Z 338" OR "acotiamide") AND ("controlled clinical trial" OR "Clinical Trial" OR "Random Allocation" OR "Double Blind Procedure" OR "single blind procedure" OR "Double-Blind*" OR "single-blind*" OR "Triple-Blind*" OR "Control Group" OR "crossover procedure" OR random* OR "Placebo" OR placebo OR trial OR groups OR crossover OR cross-over)) NOT ti=("veterinary" OR "rabbit" OR "rabbits" OR "animal" OR "animals" OR "mouse" OR "mice" OR "rodent" OR "rodents" OR "rat" OR "rats" OR "pig" OR "pigs" OR "porcine" OR "horse" OR "horses" OR "equine" OR "cow" OR "cows" OR "bovine" OR "goat" OR "goats" OR "sheep" OR "ovine" OR "canine" OR "dog" OR "dogs" OR "feline" OR "cat" OR "cats")
