# Supplementary material for: Prokinetics for the treatment of functional dyspepsia: an updated systematic review and network meta-analysis
Source: BMC Gastroenterol. 2023 Oct 31;23:370. doi: 10.1186/s12876-023-03014-9 (PMC10617220; doi:10.1186/s12876-023-03014-9)
Supplement: Supplementary file 2 — Supplementary Material 2 [file 12876_2023_3014_MOESM2_ESM.docx]

Supplementary Table 1 Characteristic of included studies

| **Study ID (author+year)** | **Diagnosis** | **Country** | **Age(years) mean±SD** | **Sex (M/F)** | **Intervention group** | | **Control group 1** | | **Control group 2** | | **Control group 3** | |
| --- | --- | --- | --- | --- | --- | --- | --- | --- | --- | --- | --- | --- |
|  |  |  |  |  | **Number of random/analysis patients** | **Drugs, dosage & treatment duration** | **Number of random/analysis patients** | **Drugs, dosage & treatment duration** | **Number of random/analysis patients** | **Drugs, dosage & treatment duration** | **Numb. Random/analysis** | **Drugs, dosage & treatment duration** |
| KUSUNOKI 2012 | FD | Japan | Total range: 20-70 Placebo：40.6±13.0 Acotiamide：40.3±13.2 | 13/24 | 21/19 | Acotiamide, 100mg × 3/day, 14-18 days | 21/18 | Placebo, 100mg × 3/day, 14-18 days | / | / | / | / |
| Chen 2004 b | FD | China | Mosapride (mean ± SD):44±12 Domperidone (mean ± SD):43±13 | 108/123 | 118/115 | Mosapride, 5 mg× 3/day, 4 weeks | 113/107 | Domperidonee, 10 mg× 3/day, 4 weeks | / | / | / | / |
| HALLERBACK 2002 | FD | Europe | Total Range: 18-75 | 204/362 | 147/140 | Mosapride, 5mg × 2/day, 6 weeks | 135/143 | Mosapride, 10mg × 2/day, 6 weeks | 154/142 | Mosapride, 7.5 mg × 2/day, 6 weeks | 152/141 | Placebo, NR, 6 weeks |
| MATSUEDA 2010-study 1 | FD | Japan | 100mg (mean ± SD): 37.5±11.5 300mg (mean ± SD): 38.6±12.8 Placebo (mean ± SD): 37.3±10.2 | 139/178 | 104/104 | Acotiamide, 100mg × 3/day, 4 weeks | 112/106 | Acotiamide, 300 mg × 3/day, 4 weeks | 107/107 | Placebo, NR, 4 weeks | / | / |
| MATSUEDA 2010-study 2 | FD | Japan | 50mg (mean ± SD)：38.9±12.4 100mg (mean ± SD)：39.1±13.4 300mg (mean ± SD): 40.6±14.2 Placebo (mean ± SD)：38.0±13.1 | 158/293 | 117/115 | Acotiamide, 50mg × 3/day, 4 weeks | 111/108 | Acotiamide, 100mg × 3/day, 4 weeks | 118/116 | Acotiamide, 300mg × 3/day, 4 weeks | 116/112 | Placebo, NR, 4 weeks |
| Zhou 2000 | FD | China | Total (range): 18-70 Itopride (mean ± SD): 42.6±12.3 Domperidone (mean ± SD): 45.8±12.8 | 72/129 | 105/100 | Itopride, 50mg × 3/day , 2 weeks | 103/101 | Domperidone, 10mg × 3/day , 2 weeks | / | / | / | / |
| Mo 2003 | FD | China | Total (Mean(Range)): 47.9 (18-70) Itopride (Mean(Range)): 48.79（30-70） Domperidone (Mean(Range)): 45.88（21-70） | 29/51 | 40/40 | Itopride, 50mg × 3/day , 2 weeks | 40/40 | Domperidone, 10mg × 3/day , 2 weeks | / | / | / | / |
| Funaki 2019 | FD | Japan | Mean ± SD: 57.3±16.8 | 7/9 | 16/16 | Acotiamide, 100mg × 3/day , 4 weeks | 16/16 | Placebo,NR, 4 weeks | / | / | / | / |
| Chen 2004 a | FD | China | Total (range): 18-65 Itopride (mean ± SD): 34.7±8.9  Domperidone (mean ± SD): 36.3±11.1 | 15/27 | 21/20 | Itopride, 50mg × 3/day , 4 weeks | 21/20 | Domperidone, 10mg × 3/day , 4 weeks | / | / | / | / |
| Zhu 2005 | FD | China | Range: 18-65 | NR | 119/119 | Itopride, 50mg × 3/day , 2 weeks | 117/117 | Domperidone, 10mg × 3/day , 2 weeks | / | / | / | / |
| Holtmann 2006 | FD | Germany | Total (range): 18~94 Placebo (mean ± SD)：49.3±15.5 Itopride (mean ± SD)： (50mg): 47.8±16.1 (100mg): 45.8±16.3 (200mg): 48.7±15.4 | 200/348 | Total: 411/406 (50mg): 139/135 (100mg): 136/135 (200mg): 136/136 | Itopride, 50/100/200mg × 3/day, 8 weeks | 143/142 | Placebo,NR, 8 weeks | / | / | / | / |
| Lin 2009 | PDS | China | Total (range): 19-65 Mosapride (range(mean ± SD)) ：19-63( 41±10) Placebo (range(mean ± SD)) : 19-65 (40±12) | 20/40 | 30/30 | Mosapride, 5mg× 3/day, 2 weeks | 30/30 | Placebo, NR, 2 weeks | / | / | / | / |
| DAVIS 1988 | FD | US | Mean(Range): 30 (18-48) | 1/15 | 9/9 | Domperidone, 20 mg× 4/day, 6 weeks | 7/7 | Placebo, NR, 6 weeks | / | / | / | / |
| Matsueda 2005 | FD | Japan | Mean: 39 | 26/39 | 33/33 | Acotiamide, 100 mg× 3/day, 4 weeks | 32/32 | Placebo, 100 mg× 3/day, 4 weeks | / | / | / | / |
| Li 2005 | FD | China | Total (range) : 18-65 Itopride (mean ± SD): 38±12; Domperidone (mean ± SD):38±12 | 94/106 | 104/100 | Itopride, 50 mg× 3/day, 4 weeks | 105/100 | Domperidone, 10 mg× 3/day, 4 weeks | / | / | / | / |
| Sun 2003 | FD | China | Range: 18-70 | NR | 120/115 | Itopride, 50mg × 3/day , 2 weeks | 120/117 | Domperidone, 10mg × 3/day , 2 weeks | / | / | / | / |
| Matsueda 2011 | FD | Japan | Placebo (mean ± SD)：37.1±9.9 Acotiamide (mean ± SD)：37.6±10.7 | 363/529 | 450/428 | Acotiamide, 100mg × 3/day , 4 weeks | 442/421 | Placebo, 100mg × 3/day , 4 weeks | / | / | / | / |
| Mierop 1979 | FD | Belgium | Total (Mean(range)): 56 (32-76)  Domperidone (Mean(range)): 55 (32-71)  Placebo (Mean(range)): 61 (37-76) | 9/23 | 17/17 | Domperidone, 20mg × 3/day, 4 weeks | 15/15 | Placebo, NR, 4 weeks | / | / | / | / |
| Van Ganse W 1978 | FD | Belgium | Mean(range): 50 ( 24–82) | 42/31 | 35/31 | Domperidone, 10 mg × 4/day, 2 weeks | 36/9 | Placebo, 10 mg × 4/day, 2 weeks | / | / | / | / |
| Bekhti A 1979 | FD | Belgium | Domperidone(median (range)): 43.5 (19–67), Placebo(median (range)): 47 (50–73) | NR | 20/13 | Domperidone, 10 mg × 4/day, 4 weeks | 20/4 | Placebo, 10 mg × 4/day, 4 weeks | / | / | / | / |
| De Loose F 1979 | FD | Belgium | Median (range): 40 (19–63) | 16/27 | 68/62 | Domperidone, 10 mg × 4/day, 2 weeks | 70/22 | Placebo, 10 mg × 4/day, 2 weeks | 68/50 | Mosapride, 10 mg × 4/day, 2 weeks | / | / |
| Van Outryve M 1979 | FD | Belgium | Domperidone(median (range)): 63 (32-80), Placebo(median (range)): 52 (25–81) | 16/22 | 16/13 | Domperidone, 20 mg × 3/day, 2 weeks | 22/11 | Placebo, 20 mg × 3/day, 2 weeks | / | / | / | / |
| Amaranpukar DN 2004 | FD | India | Itopride(mean ± SD): 45.23 ± 13.07, Mosapride(mean ± SD): 39.79 ± 10.82 | 30/30 | 30/19 | Mosapride, 5 mg × 3/day, 2 weeks | 30/28 | Itopride 50 mg × 3/day, 2 weeks | / | / | / | / |
| Talley NJ 2008 | FD | US | Itopride(mean ± SD): 42.6 ± 12.8, Placebo(mean ± SD): 43.0 ± 12.5 | Itopride:108/196, Placebo: 95/221 | 304/115 | Itopride, 100 mg × 3/day, 8 weeks | 316/112 | Placebo, 100 mg × 3/day, 8 weeks | / | / | / | / |
| Yeon-Mi Kim 2010 | FD | Korea | Mosapride(mean ± SD): 29.79 ± 7.56, Placebo(mean ± SD): 31.86 ± 11.53 | 4/24 | 14/10 | Mosapride,5 mg × 3/day, 2 weeks | 14/5 | Placebo, 5 mg × 3/day, 2 weeks | / | / | / | / |
| Du 2014 | FD | China | Cinitapride(mean ± SD):43.7± 11.9  Domperidone(mean ± SD):41.3± 12.0 | 154/229 | 191/170 | Cinitapride+Domperidone analogue, 1 mg tablet × 3/day, 4 weeks | 192/174 | Domperidone+Cinitapride analogue, 1 mg tablet × 3/day, 4 weeks | / | / | / | / |
| MORA 1993 | FD (dyskinesia and/or reflux subtypes) | Spain | Mean (range): 47 (31-65) | 7/13 | 10/10 | Metoclopramide,10 mg × 3/day, 8 weeks | 10/10 | Cinitapride, 1 mg × 3/day, 8 weeks | / | / | / | / |
| Sinha 2021 | FD-PDS | India | Acotiamide(mean ± SD): 38.1 ± 11.44  Mosapride(mean ± SD): 37.8 ± 11.56 | 5/215 | 108/107 | Acotiamide 100 mg × 3/day, 4 weeks | 112/111 | Mosapride 5 mg× 3/day, 4 weeks | / | / | / | / |

Notes: PDS: postprandial distress syndrome; FD: functional dyspepsia.

Supplementary Table 2 Outcome of included studies

| **Study ID (author+year)** | **Outcome definition** | **Measurement time** |
| --- | --- | --- |
|  |  |  |
| KUSUNOKI 2012 | Total Effective rate: We used the seven-point Likert scale to assess the change in the subjective gastrointestinal symptoms; The best condition in the seven-point Likert scale was ‘markedly improved in comparison with the baseline period'. The worst condition was ‘markedly aggravated in comparison with the baseline period'. | 14-18 days |
|  | Adverse events: Safety was evaluated by full clinical and laboratory assessment at every visit. Adverse events were recorded during the treatmentperiod, with registration of dates of onset and resolution, severity,action taken, relationship with the treatment, and outcome. | 14-18 days |
| Chen 2004 b | Early satiation effective rate: Clinical response= completely clinical response + markedly clinical response + clinical response; Individual symptoms were rated according to the following criteria: Completely clinical response: symptoms improvered by 3 grades or eliminated completely after treatment; Markedly clinical response: symptoms improved by 2 grades but not completely eliminated after treatment; Clinical response: symptoms improved by 1 grade but not completely eliminated after treatment; No effect: symptoms were progressed or no improvement. | 4 weeks |
| HALLERBACK 2002 | Effective rate: relief of dyspeptic symptoms based on the investigator’s question to the patient (‘Have your symptoms improved since you started your new medication? ) Improved %=number of cases with improvement/ total number of cases × 100% | 6 weeks |
|  | An adverse event was defined as any unfavourable or unintended sign, whether or not considered to be causally related to the study drug, and was recorded in the Case Record Form. | 6 weeks |
| MATSUEDA  2010-study 1 | Total Effective rate: patients completed a subjects global assessment of overall treatment efficacy (OTE) questionnaire. The question asked was How were your stomach symptoms during the last week in comparison to the baseline period, as assessed by the records in the paper diaries. This was scored on a 7-point Likert scale ranging from'extremely improved in comparison with the baseline period'to 'extremely aggravated in comparison with the baseline period' and 'not changed'as the middle score. patients who were 'extremely improved'or 'improved' on the OTE were considered responders. | 4 weeks |
| MATSUEDA  2010-study 2 | Total Effective rate: patients completed a subjects global assessment of overall treatment efficacy (OTE) questionnaire. The question asked was How were your stomach symptoms during the last week in comparison to the baseline period, as assessed by the records in the paper diaries. This was scored on a 7-point Likert scale ranging from'extremely improved in comparison with the baseline period'to 'extremely aggravated in comparison with the baseline period' and 'not changed'as the middle score. patients who were 'extremely improved'or 'improved' on the OTE were considered responders. | 4 weeks |
|  | Postprandial fullness elimination rate: During the 8-day baseline period and the 28-day treatment period, patients rated each of nine symptoms (upper abdominal pain, upper abdominal discomfort, postprandial fullness, upper abdominal bloating, early satiety, excessive belching, nausea,vomiting and heartburn) on a 0–3 severity scale (none, mild,moderate and severe) using paper diaries. elimination rate=score 0 | 4 weeks |
|  | Upper abdominal bloating elimination rate: During the 8-day baseline period and the 28-day treatment period, patients rated each of nine symptoms (upper abdominal pain, upper abdominal discomfort, postprandial fullness, upper abdominal bloating, early satiety, excessive belching, nausea,vomiting and heartburn) on a 0–3 severity scale (none, mild,moderate and severe) using paper diaries. elimination rate=score 0 | 4 weeks |
|  | Early satiety elimination rate: During the 8-day baseline period and the 28-day treatment period, patients rated each of nine symptoms (upper abdominal pain, upper abdominal discomfort, postprandial fullness, upper abdominal bloating, early satiety, excessive belching, nausea,vomiting and heartburn) on a 0–3 severity scale (none, mild,moderate and severe) using paper diaries. elimination rate=score 0 | 4 weeks |
|  | Adverse events (AEs) and serious AEs were recorded at each visit. In addition, vital signs, haematology, urinalysis, blood biochemistry and electrocardiography at rest were evaluated. | 4 weeks |
| Zhou 2000 | Total Effective rate: The following symptoms were observed and rated by symptom score scale before and after drug treatment: early satiety, epigastric distension, nausea, vomiting, belching, epigastric pain, epigastric discomfort, heartburn, acid regurgitation and anorexia; The symptom score was recorded according to 4 grade scale (0-3): Grade0 (0 point): asymptomatic. Grade1 (1 point) : mild but noneobvious symtoms; Grade2 (2 points): slightly severe symptoms, but did not affect work; Grade 3 (3 points): severe symptoms, difficult to persist in work. The symptom score was calculated as the total score of early satiety and abdominal distension. The symptom scores were measured before and after treatment; Clinical response rate of one symptom (%) = number of cases with complete clinical response+ markedly clinical response / total number of cases × 100%. | 2 weeks |
| Mo 2003 | Early satiation effective rate: The symptom score was recorded according to 4 grade scale (0-3): Grade0 (0 point): asymptomatic. Grade1 (1 point) : mild but noneobvious symtoms; Grade2 (2 points): slightly severe symptoms, but did not affect work; Grade 3 (3 points): severe symptoms, difficult to persist in work; Markedly clinical response: symptoms improved by at least 2 grades but not completely eliminated after treatment; Clinical response: symptoms improved by 1 grade but not completely eliminated after treatment; No effect: symptoms were progressed or no improvement; Clinical response rate of one symptom (%) = number of cases with markedly clinical response + clinical response / total number of cases × 100%。 | 2 weeks |
|  | Adverse events: Laboratory safety evaluation included blood routine, urine routine, liver and kidney function and ECG examination. The researchers closely observed the adverse reactions of drugs. | 2 weeks |
| Funaki 2019 | Responder rate based on FD score: the percentage of patients whose corresponding score decreased by 50% or more compared with that before treatment. | 4 weeks |
| Chen 2004 a | Early satiation effective rate: Individual symptoms were rated according to the following criteria: Completely clinical response: symptoms improved by 3 grades or eliminated completely after treatment; Markedly clinical response: symptoms improved by 2 grades but not completely eliminated after treatment; Clinical response: symptoms improved by 1 grade but not completely eliminated after treatment; No effect: symptoms were progressed or no improvement; Clinical response rate of one symptom (%) = number of cases with complete clinical response+ markedly clinical response + clinical response / total number of cases × 100%. | 4 weeks |
|  | Adverse events: The adverse events of drugs complained by the patients and the abnormalities of laboratory tests were assessed according to the five grades of affirmative correlation, possible relevance, affirmative irrelevance, possible irrelevance and uncertainty. Affirmative correlation and possible relevance are regarded as adverse drug reactions. | 4 weeks |
| Zhu 2005 | Early satiation rate: The following symptoms were observed and rated by symptom score scale before and after drug treatment: early satiety, epigastric distension, nausea, vomiting, belching, epigastric pain, epigastric discomfort, heartburn, acid regurgitation and anorexia; The symptom score was recorded according to 4 grade scale (0-3): Grade0 (0 point): asymptomatic. Grade1 (1 point) : mild but noneobvious symtoms; Grade2 (2 points): slightly severe symptoms, but did not affect work; Grade 3 (3 points): severe symptoms, difficult to persist in work. The symptom score was calculated as the total score of early satiety and abdominal distension. The symptom scores were measured before and after treatment; Completely clinical response: symptoms improved by 3 grades or eliminated completely after treatment; Markedly clinical response: symptoms improved by 2 grades but not completely eliminated after treatment; Clinical response: symptoms improved by 1 grade but not completely eliminated after treatment; No effect: symptoms were progressed or no improvement; Markedly clinical response rate= the number of case with markedly clinical response/ total number of cases × 100%. | 2 weeks,  4 weeks |
|  | Adverse events: Laboratory safety evaluation: blood routine; urine routine; blood electrolyte, renal function, liver function; ECG (ECG). The researchers assessed the possible association between adverse events and research drugs and drug combinations. | 4 weeks |
| Holtmann 2006 | Response rate by severity of pain and fullness: after eating as a combined end point. A positive response to treatment was prespecified as improvement by at least one grade on a five-grade scale (absent, very mild or mild, moderate, severe, or very severe) with respect to atleast one of the two symptoms (pain or fullness) and no deteriorationin the other symptom. | 8 weeks |
| Lin 2009 | Total Effective rate: The following symptoms were observed and rated by symptom score scale before and after drug treatment: early satiety, epigastric distension, nausea, vomiting, belching, epigastric pain, epigastric discomfort, heartburn, acid regurgitation and anorexia; The symptom score was recorded according to 4 grade scale (0-3): Grade0 (0 point): asymptomatic. Grade1 (1 point): mild but noneobvious symtoms; Grade2 (2 points): slightly severe symptoms, but did not affect work; Grade 3 (3 points): severe symptoms, difficult to persist in work. The symptom score was calculated as the total score of early satiety and abdominal distension. The symptom scores were measured before and after treatment; Clinical response rate of one symptom (%) = number of cases with complete clinical response+ markedly clinical response + clinical response / total number of cases × 100%. | 2 weeks |
|  | Adverse events: NR | 2 weeks |
| DAVIS 1988 | Efficient of gastric empting: Half-emptying times increased within the normal range；Delayed gastric emptying of a solid meal, defined as a half-emptying time (T1/2)>66 min | 6 weeks |
|  | Total Symptom score: Symptoms of chronic unexplained nausea, vomiting, abdominal pain, bloating, and early satiety were each given a value from 0 to 3 (none to severe) with a total possible symptom score of 0 to 15. | 6 weeks |
| Matsueda 2005 | Effective rate: Patient’s Global Symptomatic Improvement (PGSI) | 4 weeks |
|  | Adverse events: assessed by laboratory tests, adverse events (AE) and ECG | 4 weeks |
| Li 2005 | Total Effective rate: The symptom score was recorded according to 4 grade scale (0-3): Grade0 (0 point): asymptomatic. Grade1 (1 point): mild but none obvious symtoms; Grade2 (2 points): slightly severe symptoms, but did not affect work; Grade 3 (3 points): severe symptoms, difficult to persist in work. The symptom score was calculated as the total score of early satiety and abdominal distension. The symptom scores were measured before and after treatment; Clinical response rate = number of cases with complete clinical response + markedly clinical response / total number of cases x 100%; Symptoms disappear as completely clinical response; symptom improvement percentage ≥ 80% as marked improvement; 50% ≤ symptom improvement percentage < 80% as slight but clear improvement; symptom improvement percentage < 50% as no improvement; symptom improvement percentage negative as deterioration. | 1 week,  2 weeks,  4 weeks |
|  | Adverse events: The electrocardiogram, blood routine, urine routine, liver and renal function, blood electrolyte and fecal occult blood were examined before the trial and within 24 hours after the course of treatment. the number of abnormal laboratory indexes was recorded and the relationship with drugs was analyzed. | Before the trial and within 24 hours after treatment. |
| Sun 2003 | Early satiation effective rate: Total clinical response rate = number of cases with complete clinical response + markedly clinical response / total number of cases x 100%; The symptom score was recorded according to 4 grade scale (0-3): Grade0 (0 point): asymptomatic. Grade1 (1 point): mild but none obvious symtoms; Grade2 (2 points): slightly severe symptoms, but did not affect work; Grade 3 (3 points): severe symptoms, difficult to persist in work. The symptom score was calculated as the total score of early satiety and abdominal distension. The symptom scores were measured before and after treatment; Individual symptoms were rated according to the following criteria: Completely clinical response: symptoms improved by 3 grades or eliminated completely after treatment; Markedly clinical response: symptoms improved by 2 grades but not completely eliminated after treatment; Clinical response: symptoms improved by 1 grade but not completely eliminated after treatment; No effect: symptoms were progressed or no improvement. | 1 week,  2 weeks |
| Matsueda 2011 | Total Effective rate: rated each of nine symptoms (upper abdominal pain, upper abdominal discomfort, postprandial fullness, upper abdominal bloating, early satiation, excessive belching, nausea, vomiting and heartburn) on a severity scale of 0-3 (none, mild, moderate and severe) using paper diaries; a global assessment of the OTE questionnaire. This was scored in the paper diaries on a seven-point Likert scale, ranging from ‘extremely improved compared with the baseline period’, ‘improved compared with the baseline period’, ‘slightly improved compared with the baseline period’, ‘not changed’, ‘slightly aggravated compared with the baseline period’, ‘aggravated compared with the baseline period’ and ‘extremely aggravated compared with the baseline period’; achieving the two primary overall efficacy end points: “extremely improved” or“improved” were considered responders. | 8 weeks |
|  | Adverse events: results of electrocardiography, clinical laboratory tests and physical examinations were evaluated at the baseline visit, at week 4 of treatment and at week 4 of the post-treatmentfollow-up period. | 8 weeks |
| Mierop 1979 | Effective rate: the therapeutic effect obtained was individually assessed by means of a global appreciation score; Rating possibilities for the global appreciation were: excellent (rapid and sustained disappearance of the cardinal symptoms), good (marked improvement), moderate (slight but clear improvement), poor (no or almost no improvement). Excellent+good=efficacy; Efficacy%=（excellent+good）/total*100% | 2 weeks,  4 weeks |
| Van Ganse W 1978 | Total effective rate: NR | NR |
| Bekhti A 1979 | Total effective rate: NR | NR |
| De Loose F 1979 | Total effective rate: NR | NR |
| Van Outryve M 1979 | Total effective rate: NR | NR |
| Amaranpukar DN 2004 | Total effective rate: NR | NR |
| Talley NJ 2008 | Total effective rate: NR | NR |
| Yeon-Mi Kim 2010 | Total effective rate: NR | NR |
| Du 2014 | Total effective rate: FD symptoms mean score:The total symptom score consisted of the sum of 3 symptoms（early satiation,postprandial fullness, bloating）. Patients assessed severity of their own symptoms using a 5-point Likert scale: none (0), mild (1), moderate (2), severe (3), and extremely (4); Symptom frequency was also assessed using a 5-point Likert scale: none (0), <1 d/wk (1), 1 d/wk (2), >1 d/wk (3), and everyday (4)；Total score was obtained by summing each symptom severity multiplied by its frequency, and a value of 48 points represented the most severe symptom intensity. | 4 weeks |
|  | Adverse events: potential cardiovascular side effects were evaluated using 12-lead ECG after 2 and 4 weeks and at additional times, if necessary. The incidence rates of adverse events (AEs) and adverse drug reactions (ADRs) were also evaluated. | 4 weeks |
| MORA 1993 | Total Effective rate: At the end of each treatment phase, an overall assessment was conducted and the results were compared with those of the previous phase. The evaluation results were divided into the following levels: good = symptoms disappeared; general = symptoms improved, but some symptoms still existed; poor = no improvement; Evaluation symptoms include: postprandial upper abdominal flatulence, flatulence, epigastric pain, heartburn, reflux, nausea, vomiting, anorexia and abnormal defecation per week. According to the frequency and severity of the symptoms, it is divided into the following levels: 0 = asymptomatic; 1 = mild (patients should be reminded of symptoms) with low frequency (1 to 3 days a week); 2 = moderate intensity (patients are aware of symptoms but can perform regular activities) and moderate frequency (4 to 5 days a week); 3 = severe (patients can only occasionally carry out daily activities) and have a higher frequency (every day of the week). | 8 weeks |
| Sinha 2021 | Total effective rate: Responder rates based on overall treatment effect by using a seven-point Likert scale at the end of treatment visit was the primary efficacy endpoint. On the OTE scale, patients with “extremely improved” or “improved” were considered as responders.  Safety variables included all treatment-emergent clinical and laboratory adverse events. |  |

Notes: FSSG: the modified frequency scale for the symptoms of gastroesophageal reflux disease questionnaire; GSRS: gastrointestinal symptom rating scale.

Supplementary Table 3 Description results of specific adverse event in Acotiamide, Domperidone, Itopride and Cinitapride.

| Study ID (author + year) | Drug | Outcome | Follow-up time | Risk |
| --- | --- | --- | --- | --- |
|  |  |  |  | Event/Total |
| KUSUNOKI 2012 | Acotiamide | Increase of blood prolactin | 14-18 days | 2/21^c^ |
|  |  | Increase of alanine aminotransferase | 14-18 days | 1/21 |
|  |  | Increase of blood potassium | 14-18 days | 1/21 |
|  |  | Increase of blood hilimhin | 14-18 days | 0/21 |
|  |  | Increase of lactic acid dehydrogenase | 14-18 days | 0/21 |
|  |  | Urine glucose positivity | 14-18 days | 0/21 |
|  |  | Urine protein positivity | 14-18 days | 0/21 |
|  |  | Taste disturbance | 14-18 days | 0/21 |
| Li 2005 | Domperidone | Serious AE^a^ | 4 weeks | 1/101 |
| Chen 2004a | Domperidone | lower limb skin rash | 5 days | NR/20 |
|  |  | mild lower abdominal pain | 1 week | 1/20 ^c^ |
|  |  | expressive galactorrhea | 5 days | 1/20 ^c^ |
| DAVIS 1988 | Domperidone | Expressive galactorrhea and bilateral breast tenderness  (only bilateral breast tenderness in the placebo group) - drug-related | 6 weeks | 4/9 ^c^ |
|  |  | Constipation - drug-related | 6 weeks | 1/9 ^c^ |
|  |  | Decreased appetite - drug-related | 6 weeks | NR/9 |
|  |  | Hyperprolactinemia - drug-related | After 6 weeks | 9/9 ^c^ |
| Du 2014 | Domperidone | AE- non drug related | 8 weeks | 16/192 ^c^ |
| Li 2005 | Itopride | Serious AE^b^ | 4 weeks | 0/100 |
| Chen 2004a | Itopride | lower limb skin rash | 5 days | 1/20 ^c^ |
|  |  | mild lower abdominal pain | 1 week | 2/20 ^c^ |
|  |  | expressive galactorrhea | 5 days | NR/20 |
| Du 2014 | Cinitapride | AE- non drug related | 8 weeks | 6/191 |

Notes: a, Only one serious adverse event was reported: an intervertebral disc herniation occurred in one of the 450 patients (0.2%) treated with Domperidone, judged as ‘not related’; b, Only one case in the control group developed moderate and severe abdominal pain, diarrhea, intestinal tinnitus and dizziness at 1 day after treatment, stopped taking the drug for 2 days, and all the symptoms disappeared 3-7 days after drug withdrawal; c, the specific adverse events was ≥5%; AE, adverse events.

Supplementary Table 4 SUCRA of each treatment for total efficacy rate

| Treatment | SUCRA |
| --- | --- |
| Metoclopramide | 0.979 |
| Cinitapride | 0.821 |
| Domperidone | 0.632 |
| Acotiamide | 0.473 |
| Itopride | 0.393 |
| Mosapride | 0.2011 |
| Placebo | 0.0003 |

Notes: SUCRA: surface under the cumulative ranking curve. Higher value means higher probability of an effective drug.

Supplementary Table 5 SUCRA of each treatment for total adverse events

| Treatment | SUCRA |
| --- | --- |
| Cinitapride | 0.935 |
| Itopride | 0.341 |
| Domperidone | 0.225 |

| Treatment | SUCRA |
| --- | --- |
| Placebo | 0.745 |
| Mosapride | 0.508 |
| Acotiamide | 0.247 |

Notes: SUCRA: surface under the cumulative ranking curve. Higher value means higher probability of a safer drug. Due to insufficient reporting of this outcome, there were two treatment loops: cinitapride-itropride-domperidone loop and acotiamide-mosapride-placebo loop. Therefore, there were one ranking result within each loop. Higher value means higher probability of a safer drug.

Supplementary Table 6 SUCRA of each treatment for drug-related adverse events related to prokinetics

| Treatment | SUCRA |
| --- | --- |
| Domperidone | 0.691 |
| Cinitapride | 0.687 |
| Placebo | 0.512 |
| Itopride | 0.328 |
| Acotiamide | 0.282 |

`Notes: SUCRA: surface under the cumulative ranking curve. Higher value means higher probability of a safer drug.
