# Supplementary material for: Prokinetics for the treatment of functional dyspepsia: an updated systematic review and network meta-analysis
Source: BMC Gastroenterol. 2023 Oct 31;23:370. doi: 10.1186/s12876-023-03014-9 (PMC10617220; doi:10.1186/s12876-023-03014-9)
Supplement: Supplementary file 4 — Supplementary Material 4 [file 12876_2023_3014_MOESM4_ESM.docx]

**Supplementary Figures**


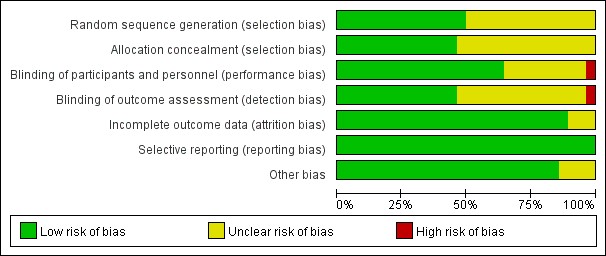


Supplementary Figure 1 Risk of bias graph


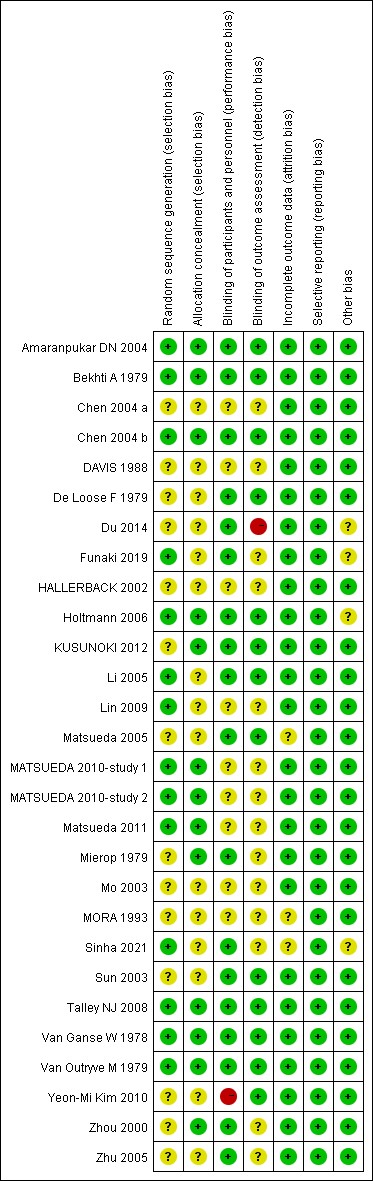


Supplementary Figure 2 Risk of bias summary


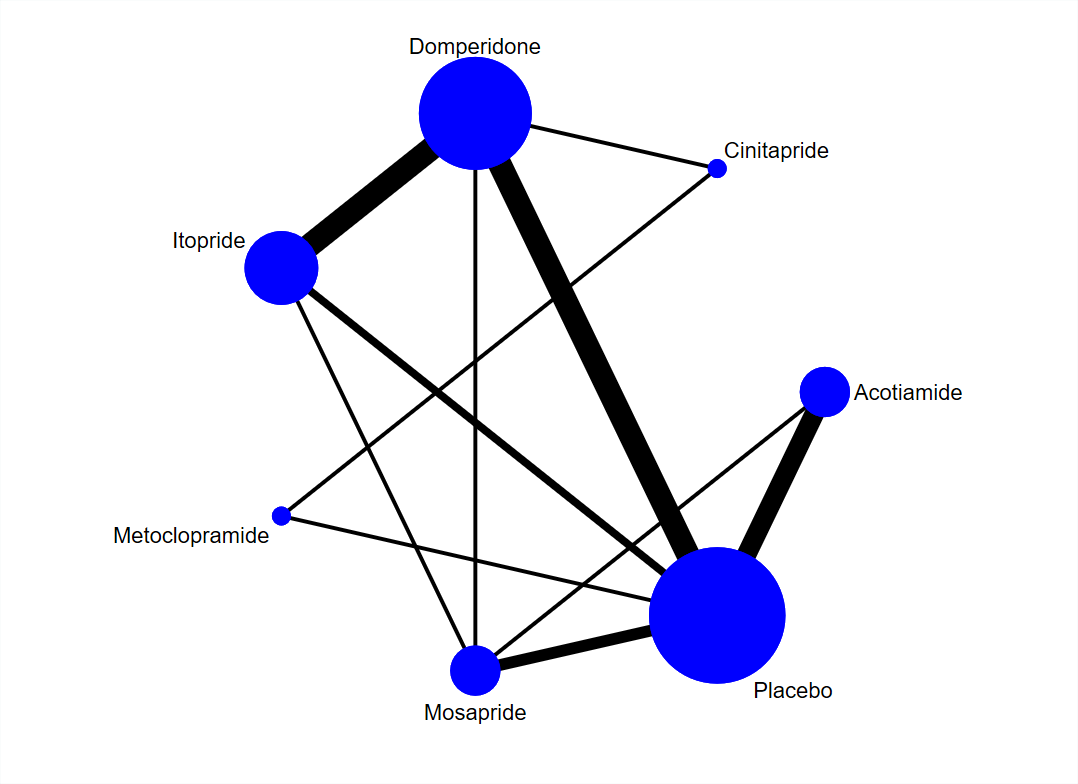


Supplementary Figure 3 Network plot of relevant studies on total efficacy rate

Notes: Nodes represent the prokinetic drugs and lines represent the direct comparisons. The size of each node indicates the overall number of participants receiving each prokinetic drug, and the line thickness between the nodes indicates the number of studies comparing two drugs.


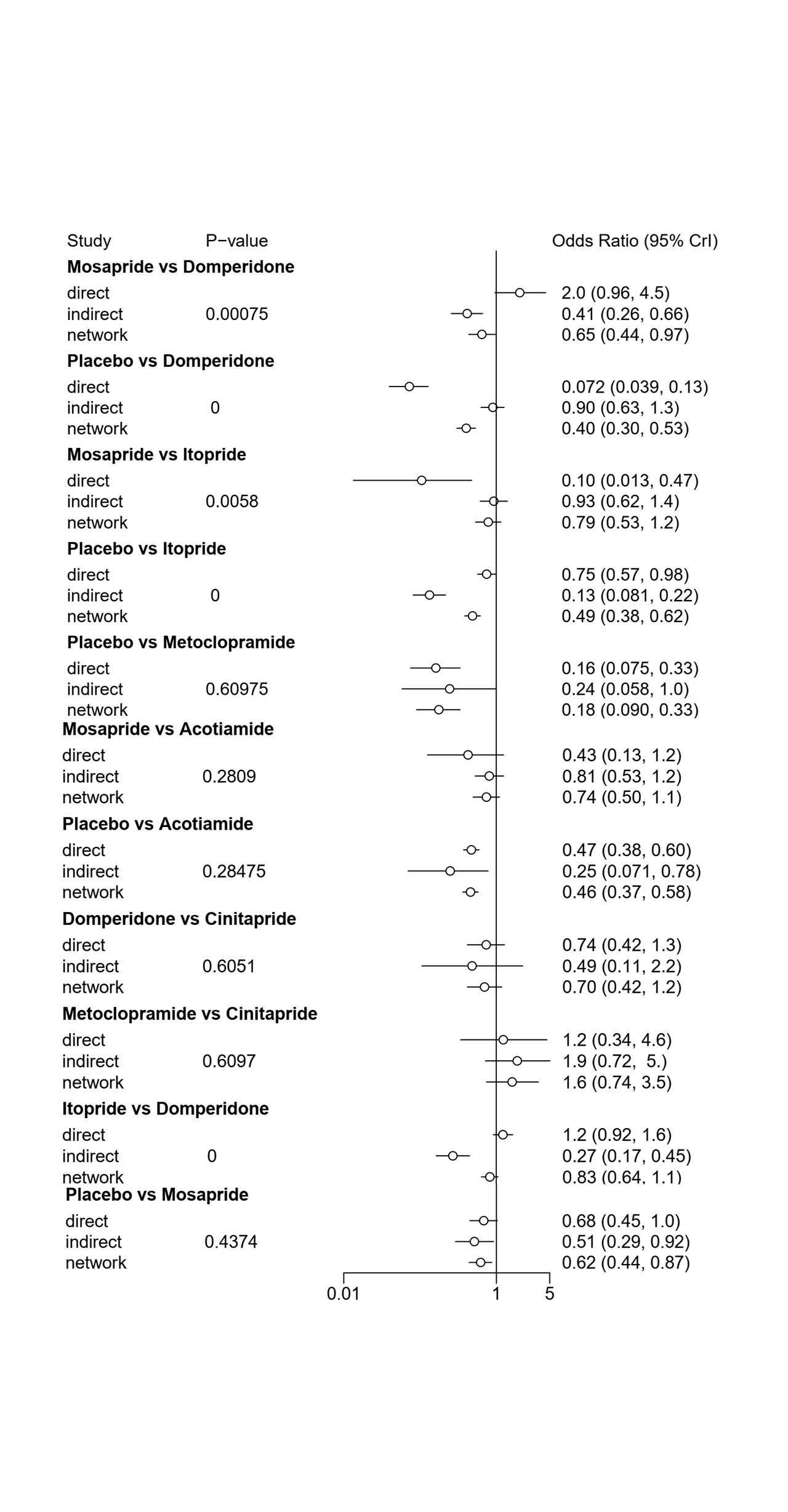


Supplementary Figure 4 Nodesplit forest plot of total efficacy rate


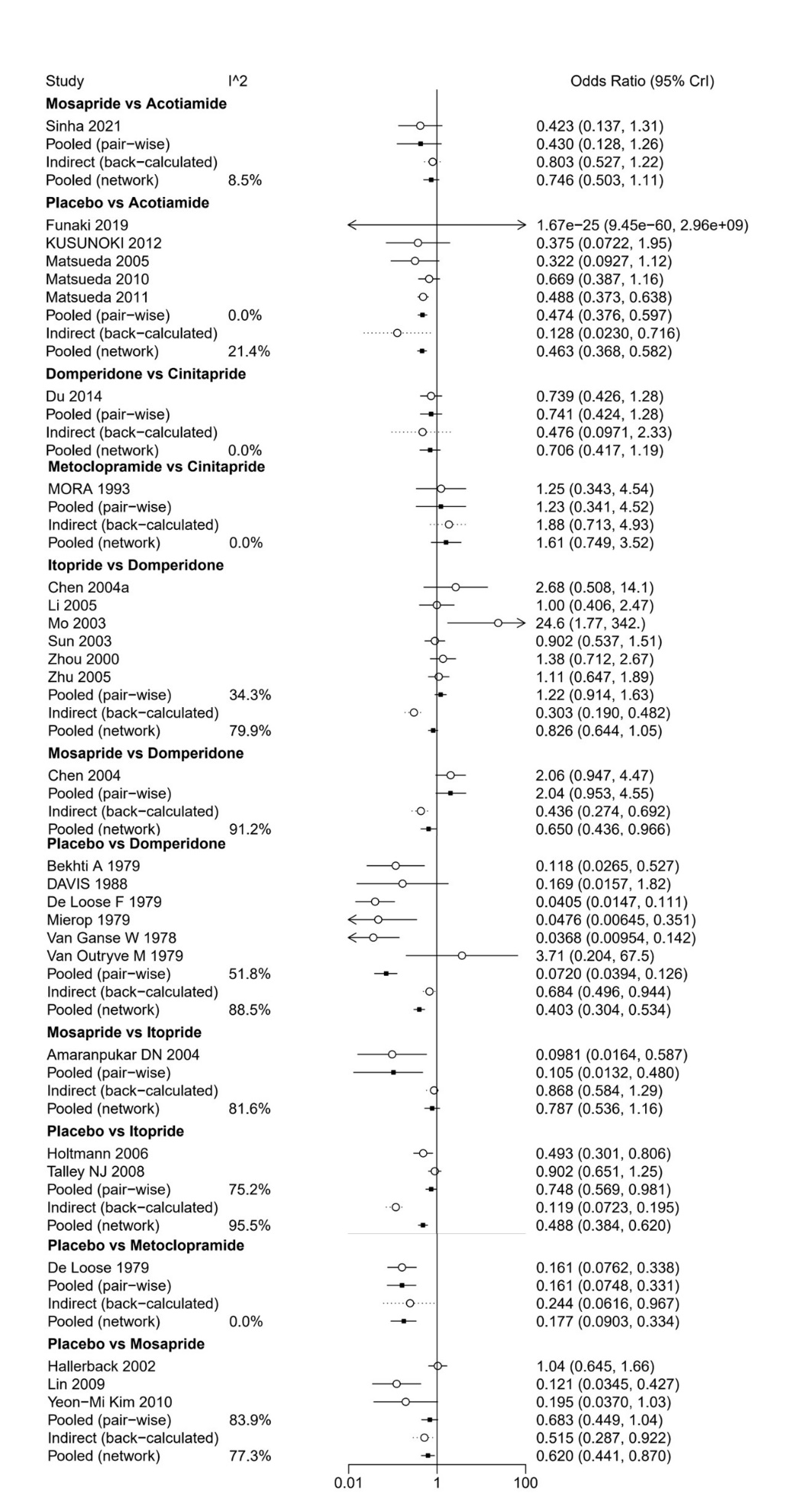


Supplementary Figure 5 Pairwise meta-analysis forest plot of total therapeutic efficacy rate


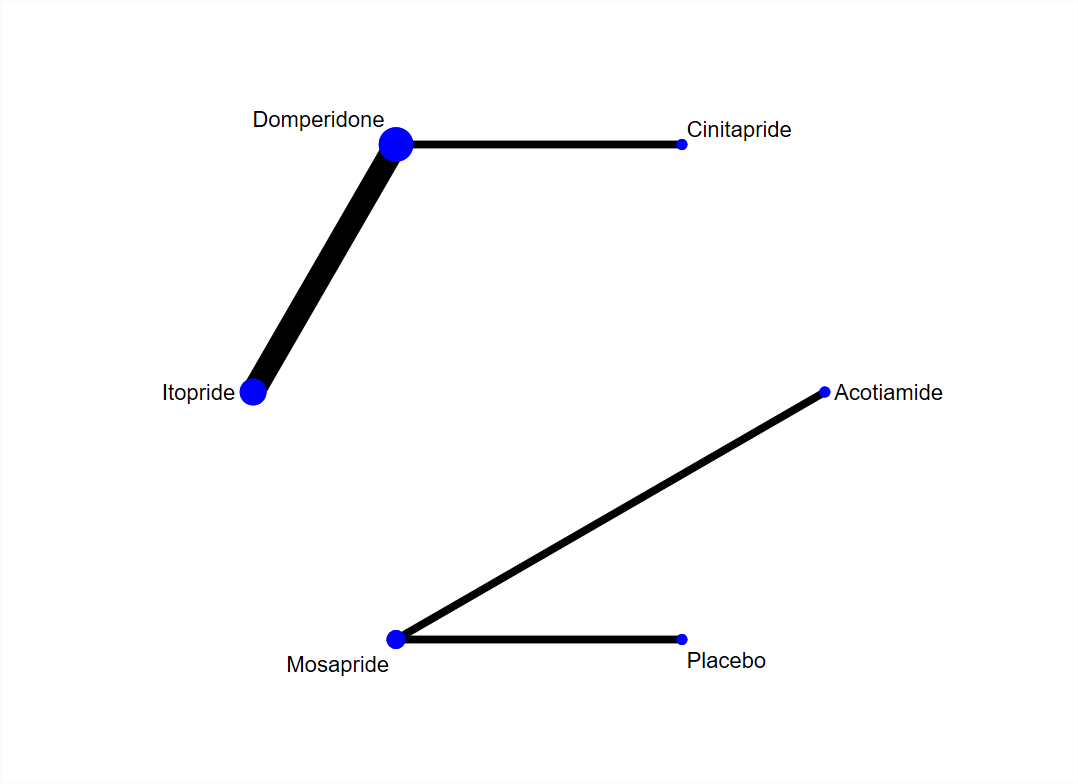


Supplementary Figure 6 Network plot of total adverse events rate from relevant studies

Notes: Nodes represent prokinetic drugs, and lines represent the direct comparisons. The size of each node indicates the overall number of participants receiving each prokinetic drug, and line thickness between the nodes indicates the number of studies comparing two drugs


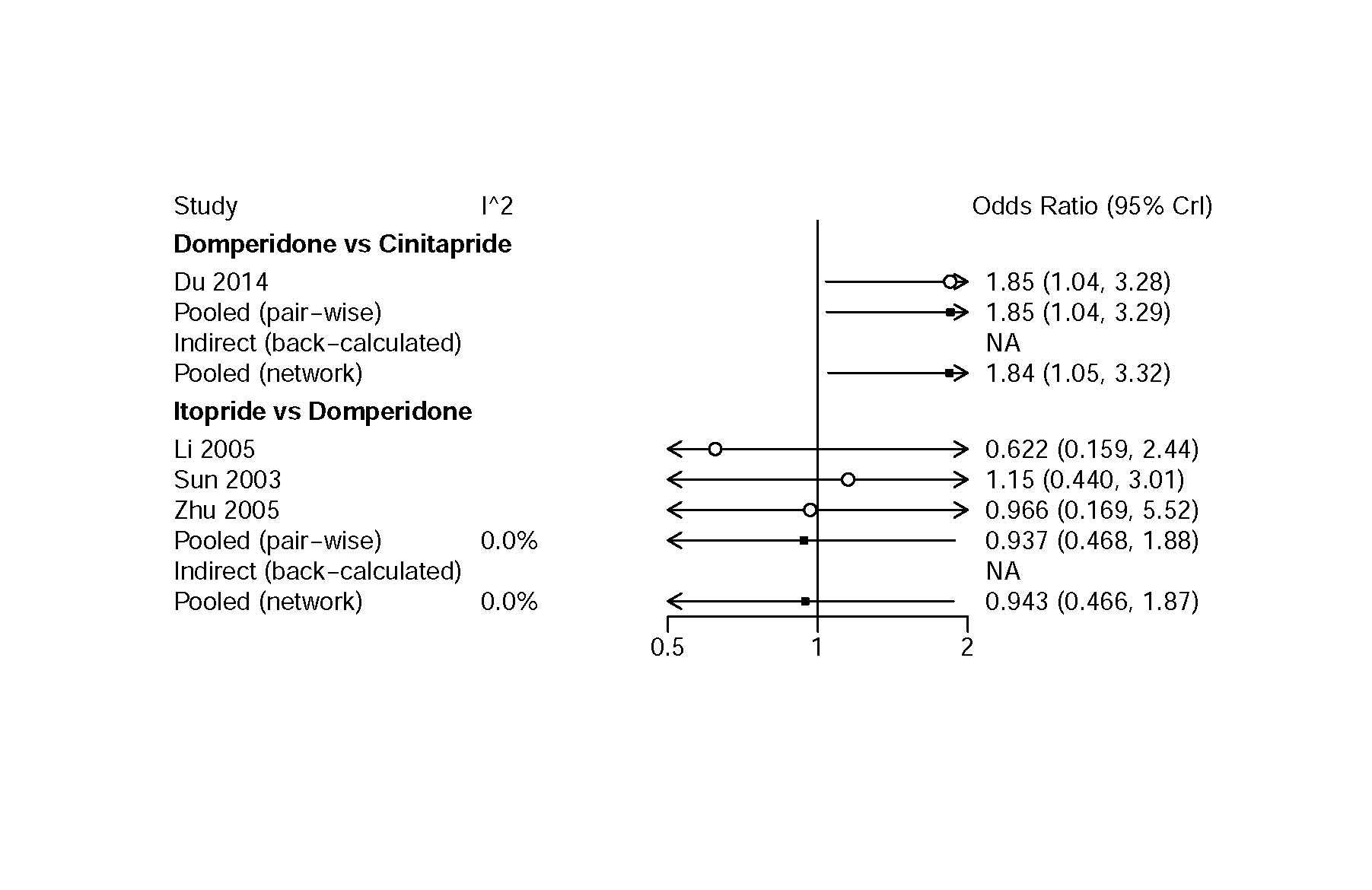


Supplementary Figure 7 Pairwise meta-analysis forest plot of total adverse events rate.

Note: As there is only one study comparing mosapride with acotiamide and placebo reporting total adverse events respectively, meta-analysis between mosapride-acotiamide-placebo was not performed.


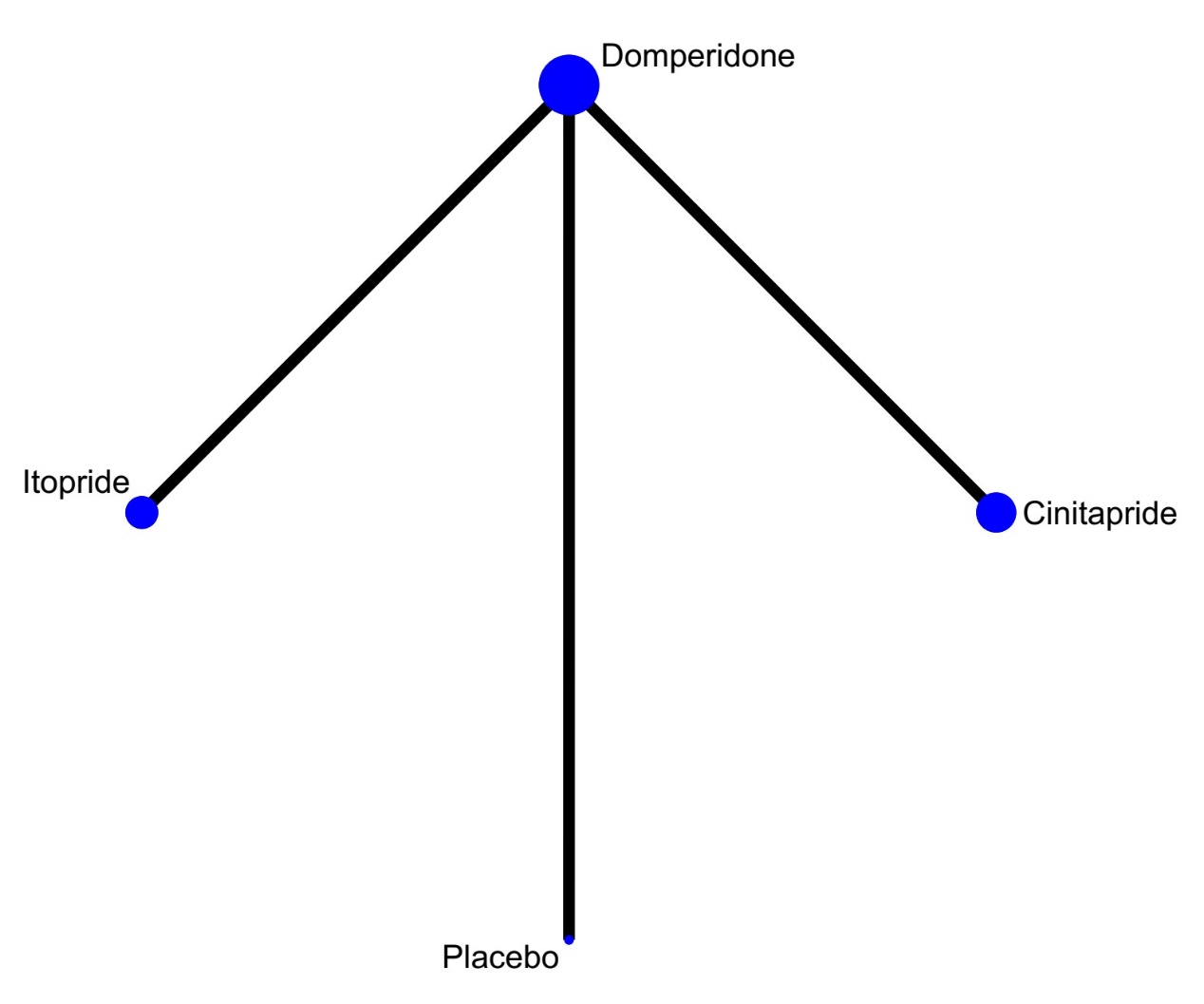


Supplementary Figure 8 Network plot of relevant studies on drug-related adverse events rate
