# Supplementary material for: Prokinetics for the treatment of functional dyspepsia: an updated systematic review and network meta-analysis
Source: BMC Gastroenterol. 2023 Oct 31;23:370. doi: 10.1186/s12876-023-03014-9 (PMC10617220; doi:10.1186/s12876-023-03014-9)
Supplement: Supplementary file 5 — Supplementary Material 5 [file 12876_2023_3014_MOESM5_ESM.docx]

Excluded studies list

1. No outcome data[1-9]

2. Not eligible intervention[10]

3. Not eligible outcome data[11,12]

4. Not eligible patients[13]

5. Not RCT[14-16]

6. Only abstract[17-21]

7. Systematic review[22-28]

Excluded studies list:

1. Nakamura K, Tomita T, Oshima T, et al. A double-blind placebo controlled study of acotiamide hydrochloride for efficacy on gastrointestinal motility of patients with functional dyspepsia. J Gastroenterol 2016;52:602-610.

2. Yoon H, Lee DH, Lee YH, et al. Multi-center, randomized, active-controlled, double-blind, non-inferiority, phase 3 clinical trial to evaluate the efficacy and safety of UI05MSP015CT in functional dyspepsia (Mars study). Gastroenterology 2017;152:S936.

3. Bang CS, Baik GH, Suk KT, et al. Prokinetics for the treatment of functional dyspepsia: A bayesian network meta-analysis. United European Gastroenterology Journal 2016;4:A679.

4. Kountouras J, Gavalas E, Papaefthymiou A, et al. Trimebutine Maleate Monotherapy for Functional Dyspepsia: A Multicenter, Randomized, Double-Blind Placebo Controlled Prospective Trial. Medicina (Kaunas) 2020;56.

5. Abid S, Jafri W, Zaman MU, et al. Itopride for gastric volume, gastric emptying and drinking capacity in functional dyspepsia. World J Gastrointest Pharmacol Ther 2017;8:74-80.

6. Carbone F, Vandenberghe A, Holvoet L, et al. Validation of the Leuven Postprandial Distress Scale, a questionnaire for symptom assessment in the functional dyspepsia/postprandial distress syndrome. Aliment Pharmacol Ther 2016;44:989-1001.

7. Chen SL, Ji JR, Xu P, et al. Effect of domperidone therapy on nocturnal dyspeptic symptoms of functional dyspepsia patients. World J Gastroenterol 2010;16:613-617.

8. Banani SJ, Lankarani KB, Taghavi A, et al. Comparison of metoclopramide oral tablets and solution in treatment of dysmotility-like dyspepsia. Am J Health Syst Pharm 2008;65:1057-1061.

9. Della Coletta M, Andreozzi P, Zito FP, et al. Mental stress and meal induced sympathetic activity in postprandial distress syndrome patients: Effect of domperidone. Digestive and Liver Disease 2014;46:S7.

10. Hojo M, Nagahara A, Asaoka D, et al. A Randomized, Double-Blind, Pilot Study of the Effect of Famotidine on Acotiamide Treatment for Functional Dyspepsia. Digestion 2017;96:5-12.

11. Carbone F, Vandenberghe A, Holvoet L, et al. A double-blind randomized, multicenter, placebo-controlled study of itopride in functional dyspepsia postprandial distress syndrome. 2022;34:e14337.

12. Ctri. A Clinical study to evaluate the effect of ASFP001/21 and SMXP001/22 on improvement in quality of life in healthy volunteers with mild to moderate functional dyspepsia symptoms. https://trialsearchwhoint/Trial2aspx?TrialID=CTRI/2022/07/044075 2022.

13. Van den Houte K, Carbone F, Pauwels A, et al. Influence of itopride and domperidone on gastric tone and on the perception of gastric distention in healthy subjects. Neurogastroenterol Motil 2019;31:e13544.

14. Singh H, Bala R, Kaur K. Efficacy and tolerability of levosulipride, domperidone and metoclopramide in patients with non-ulcer functional dyspepsia: a comparative analysis. J Clin Diagn Res 2015;9:Fc09-12.

15. Pittayanon R, Yuan Y, Bollegala NP, et al. Prokinetics for functional dyspepsia. Cochrane Database of Systematic Reviews 2017;2017.

16. Kountouras J, Doulberis M, Papaefthimiou A, et al. Gastroesophageal reflux disease, irritable bowel syndrome and functional dyspepsia as overlapping conditions: Focus on effect of trimebutine. Annals of Gastroenterology 2019;32:318.

17. Ctri. Acotiamide in functional dyspepsia. https://trialsearchwhoint/Trial2aspx?TrialID=CTRI/2022/08/044700 2022.

18. Van Den Houte K, Carbone F, Pauwels A, et al. Impact of itopride and domperidone on the sensitivity of gastric distention and gastric accommodation in healthy volunteers. United European Gastroenterology Journal 2018;6:A506.

19. Van Den Houte K, Carbone F, Pauwels A, et al. Influence of itopride and domperidone on gastric tone and on the perception of gastric distention in healthy subjects. Neurogastroenterology and Motility Conference: 3rd Meeting of the Federation of Neurogastroenterology and Motility and Postgraduate Course on Gastrointestinal Motility, FNM 2018;30.

20. Yoon H, Lee DH, Lee YH, et al. Efficacy and Safety of UI05MSP015CT in Functional Dyspepsia: A Randomized, Controlled Trial. Gut Liver 2018;12:516-522.

21. Nakamura K, Tomita T, Oshima T, et al. Correction to: A double-blind placebo controlled study of acotiamide hydrochloride for efficacy on gastrointestinal motility of patients with functional dyspepsia. J Gastroenterol 2021;56:191.

22. Ford AC, Moayyedi P. Systematic review and network meta-analysis: efficacy of drugs for functional dyspepsia. 2021;53:8-21.

23. Shrestha DB, Budhathoki P, Subedi P, et al. Acotiamide and Functional Dyspepsia: A Systematic Review and Meta-Analysis. Digestive diseases and sciences 2021;13:e20532.

24. Bang CS, Kim JH, Baik GH, et al. Mosapride treatment for functional dyspepsia: a meta-analysis. J Gastroenterol Hepatol 2014;30:28-42.

25. Vijayvargiya P, Camilleri M, Chedid V, et al. Effects of Promotility Agents on Gastric Emptying and Symptoms: A Systematic Review and Meta-analysis. Gastroenterology 2019;156:1650-1660.

26. Ford AC, Moayyedi P, Black CJ, et al. Systematic review and network meta-analysis: efficacy of drugs for functional dyspepsia. Aliment Pharmacol Ther 2020;53:8-21.

27. Yang YJ, Bang CS, Baik GH, et al. Prokinetics for the treatment of functional dyspepsia: Bayesian network meta-analysis. BMC Gastroenterol 2017;17:83.

28. Everhart K, Lacy B. Functional dyspepsia and prokinetics: A systematic review of gastric emptying, health-related quality of life and symptom severity. American Journal of Gastroenterology 2014;109:S645-S646.
